# An amphioxus orthologue of the estrogen receptor that does not bind estradiol: Insights into estrogen receptor evolution

**DOI:** 10.1186/1471-2148-8-219

**Published:** 2008-07-25

**Authors:** Mathilde Paris, Katarina Pettersson, Michael Schubert, Stephanie Bertrand, Ingemar Pongratz, Hector Escriva, Vincent Laudet

**Affiliations:** 1 Institut de Génomique Fonctionnelle de Lyon, Molecular Zoology team, Université de Lyon, Ecole Normale Supérieure de Lyon, Université Lyon 1, CNRS, INRA, Institut Fédératif 128 Biosciences Gerland Lyon Sud, France; 2 Karolinska Institutet, Department of Biosciences and Nutrition, S-141 57 Huddinge, Sweden; 3 CNRS, UMR 7628, Modèles en Biologie Cellulaire et Evolutive, Observatoire océanographique, F-66651, Banyuls/mer, France

## Abstract

**Background:**

The origin of nuclear receptors (NRs) and the question whether the ancestral NR was a liganded or an unliganded transcription factor has been recently debated. To obtain insight into the evolution of the ligand binding ability of estrogen receptors (ER), we comparatively characterized the ER from the protochordate amphioxus (*Branchiostoma floridae*), and the ER from lamprey (*Petromyzon marinus*), a basal vertebrate.

**Results:**

Extensive phylogenetic studies as well as signature analysis allowed us to confirm that the amphioxus ER (amphiER) and the lamprey ER (lampER) belong to the ER group. LampER behaves as a "classical" vertebrate ER, as it binds to specific DNA Estrogen Responsive Elements (EREs), and is activated by estradiol (E_2_), the classical ER natural ligand. In contrast, we found that although amphiER binds EREs, it is unable to bind E_2 _and to activate transcription in response to E_2_. Among the 7 natural and synthetic ER ligands tested as well as a large repertoire of 14 cholesterol derivatives, only Bisphenol A (an endocrine disruptor with estrogenic activity) bound to amphiER, suggesting that a ligand binding pocket exists within the receptor. Parsimony analysis considering all available ER sequences suggest that the ancestral ER was not able to bind E_2 _and that this ability evolved specifically in the vertebrate lineage. This result does not support a previous analysis based on ancestral sequence reconstruction that proposed the ancestral steroid receptor to bind estradiol. We show that biased taxonomic sampling can alter the calculation of ancestral sequence and that the previous result might stem from a high proportion of vertebrate ERs in the dataset used to compute the ancestral sequence.

**Conclusion:**

Taken together, our results highlight the importance of comparative experimental approaches vs ancestral reconstructions for the evolutionary study of endocrine systems: comparative analysis of extant ERs suggests that the ancestral ER did not bind estradiol and that it gained the ability to be regulated by estradiol specifically in the vertebrate lineage, before lamprey split.

## Background

Hormone signaling is a very important feature in metazoans, allowing communication between cells or organs within the organism. Two components of these signaling systems are of particular importance, the hormone and its receptor. The nuclear hormone receptor (NR) superfamily includes ligand dependent transcription factors that play a central role in various physiological processes as diverse as reproduction, development, and control of homeostasis [1,2]. They share a common structural organization and exhibit a highly conserved DNA binding domain (DBD) and a moderately conserved ligand-binding domain (LBD). Some members of this superfamily are liganded receptors (24 among the 48 genes encoding NRs in the human genome) but many lack identified ligand and are therefore called "orphan" [3]. Some orphan receptors are 'true' orphans in the sense that they do not possess a *bona fide *ligand-binding pocket (LBP), like the members of the NR4 subfamily (for instance, NURR1, DHR38 or NGFI-B. For review, see [4]), and are regulated by other mechanisms [4]. Alternatively, the crystal structures of several orphan receptors such as HNF4 were found to have a phospholipid constitutively bound to a large ligand binding pocket [5,6]. The functional and evolutionary implications of these constitutive ligands remain discussed. Other orphan nuclear receptors have a ligand binding pocket and thus have the potential to bind compounds. It is still not known whether those receptors have natural ligands, still to be discovered. Undoubtedly, the existence of such orphan receptors with physiological or developmental activities constitutes both a major challenge for understanding nuclear receptor evolution and a potential opportunity for pharmacology [1].

The existence of orphan and liganded members in the NR family raises the question of the evolution regarding their ligand binding ability. Whether the ancestral NR was liganded or orphan and more generally how NR ligand binding ability evolved has been recently debated [7-14]. In general, it is still unclear if there is a correlation between the evolution of the hormone repertoire and NRs. Moreover the mechanisms underlying this coevolution are of particular interest [7,12,15-19].

Among the scenarios of NR evolution that have been proposed, one suggests that the ancestral NR was a ligand-independent transcription factor which acquired the ability to be regulated by ligands several times during evolution [7,18-20]. This hypothesis was based on the observation that compounds of similar chemical nature bind to divergent NRs and on the contrary compounds of very different nature bind to closely related receptors. For instance, orphan receptors are found in all families of NRs, and steroid receptors are not monophyletic but are located in two different subfamilies within the NR superfamily: the ecdysteroid as well as the sex steroid receptors. Interestingly, the evolution of sex steroid hormone receptors has also been used as an argument for an alternative hypothesis, the ligand exploitation model [8,11] (for an alternative view, see [21,22]). Phylogenetic trees show that sex steroid hormone receptors are grouped with ERRs as the NR3 subfamily, following the official nomenclature [23]. They contain receptors that bind estradiol (ERs), that form the NR3A group as well as mineralocorticoids (MRs), glucocorticoids (GRs), progesterone (PRs), and androgen (ARs) that form the NR3C group. All known ligands in this subfamily can be seen as variations around the archetypical sterol skeleton. Consequently, Thornton et al. suggested in the ligand exploitation model that the ancestral steroid receptor was a high affinity estradiol receptor [8,11] and the other steroid receptors that originated later on, experienced, following gene duplication, shifts in their binding affinities to eventually bind to their extant ligand. The model in fact suggests that the newly duplicated receptors (here NR3C) exploit as ligands chemical species that serve as intermediary compounds in the "ancestral ligand" synthesis pathway (here the estradiol synthesis pathway) [8]. According to this view, orphan receptors, like ERRs, secondarily lost the ability to have their activity regulated by a ligand and became orphan. Interestingly, within the NR3 family, two receptor subfamilies, ERRs and ERs, appear to be ancient since they are found in a wide variety of metazoans including deuterostomes and protostomes, whereas, up to now, MRs, GRs, PRs and ARs have been found only in vertebrates. The only non-vertebrate ERs that have been described so far were from mollusks and were shown to be unable to bind estradiol [11,12,24-26]. Since the ligand exploitation model implies an ancestral estradiol-binding ER and since all liganded ER found so far come from vertebrates, and to improve taxonomic sampling, the ER orthologues from the basal vertebrate lamprey and the invertebrate chordate amphioxus were characterized here. Indeed, lamprey and amphioxus are located at key positions in the chordate phylum [27-30]. Moreover, amphioxus (*Brachiostoma floridae*) is much less derived than urochordates in its morphology as well as in its genome organization [30]. Indeed, amphioxus and vertebrates share a similar general body plan whereas urochordate morphology is more derived. For instance, during metamorphosis of some urochordates, the tadpole-like larva transforms into an adult that looks so different that it was first considered as a mollusk [31]. Moreover the urochordate genome is fast evolving [27], with for instance the loss of the clustering of the hox genes [32]. There is no ER in the sequenced genome of *Ciona intestinalis *[33] or in the sea urchin [34], one ER was previously cloned in lamprey [8], only one ER was found in the amphioxus genome [35]. These reasons make lamprey and amphioxus excellent models to study the evolution of estrogen signaling pathway at the origin of vertebrates. In this study, we cloned the unique ER from amphioxus (amphiER) and characterized it, as well as the previously cloned but uncharacterized lamprey ER (lampER). AmphiER is an orphan receptor, showing no affinity to the estrogen hormone estradiol, when in contrast, the lamprey ER behaves as a "classical" vertebrate ER. As no ER from invertebrates studied so far binds estradiol, we propose that the ancestral ER (and the ancestral steroid receptor) was not a receptor for estradiol and gained later on during evolution the ability to bind the hormone.

## Results

### Cloning of the ER from amphioxus (amphiER)

**Figure 1:**
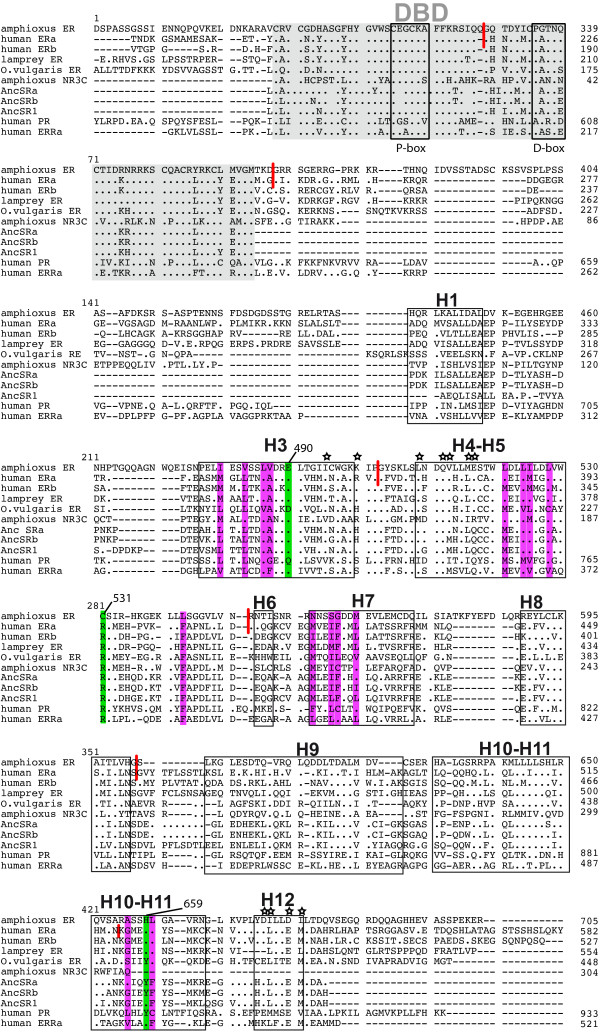
**Sequence alignment of several ERs including the amphioxus ER and the lamprey ER, as well as ancestral steroid receptors**. The DBD is highlighted with light grey. The 12 helices from the LBD are indicated, based on the known 3D structure of human ERα [49]. Amino acids from human ERα making direct hydrogen bonds with E_2 _are indicated in green. Amino acids making hydrophobic bonds with E_2 _are highlighted in purple. Amino acids known to be involved in co-activator interaction have been indicated with a star on top of each site [55]. The more divergent A/B domain as well as the F domain have been omitted from the alignment. However, the numbering of the sites along the alignment starts at the beginning of each protein. The exon-intron limits of amphiER and humanERα have been indicated with small red strokes. The sequences of AncSRa and AncSRb have been inferred in this study. The sequence of AncSR1 was retrieved from a previous analysis [11].

**Figure 2:**
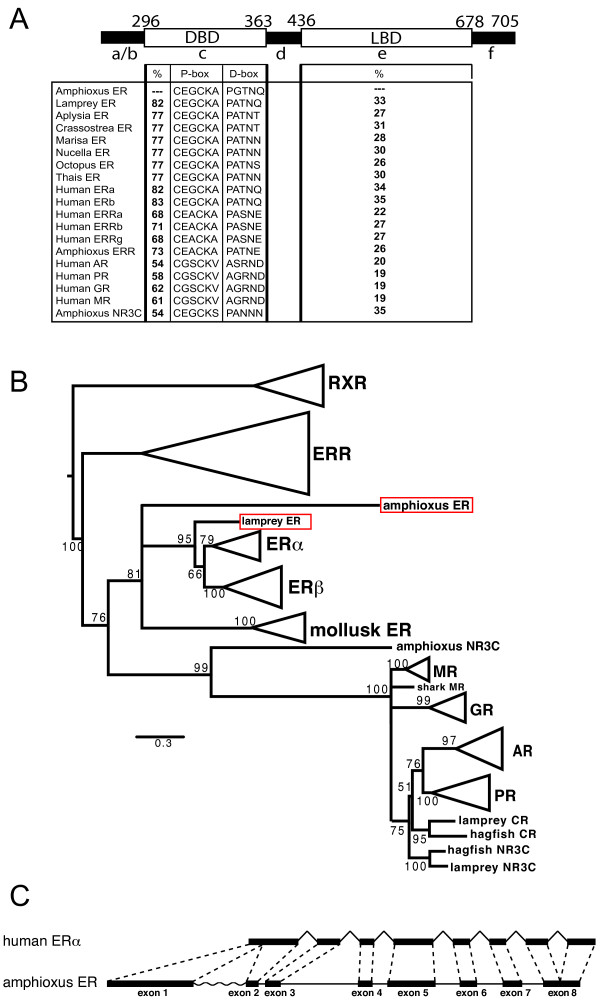
**There is a single ER in amphioxus**. (A) Schematic representation of the different domains of amphiER. Percent identity of the amphioxus ER with other sequences from the NR3 subfamily in the DNA- and ligand-binding domains is indicated. Amino acid sequence of the highly conserved P-box and D-box in the DBD are shown. (B) Maximum likelihood (ML) tree obtained from the analysis of the amino acid sequences of the DBD and the LBD of a wide range of NR3 under a JTT+γ +i model. Bootstrap percentages obtained after 1,000 ML replicates are shown above selected branches. Scale bar indicates number of changes per site. The tree was rooted by selected RXR sequences. (C) The exon-intron structure of amphiER is conserved with that of human ERα, except two minor differences: the first human exon corresponds to the first two amphioxus exons and the last two human exons correspond to the last amphioxus exon.

Using degenerate primers designed to match motifs in the most conserved part of vertebrate ERs in the DNA binding domain, a single gene fragment from total RNA of an adult *Branchiostoma floridae *was amplified, cloned and sequenced. Rapid amplification of cDNA ends (RACE) was utilized to obtain the full-length cDNA. From this sequence, a new set of specific primers were designed and used to amplify the full length open reading frame of this gene. The obtained cDNA [GenBank: ACF16007] is 2118 bp long and encodes a 705 aa long putative protein (Figure 1) that harbors the classical features of an ER with the 5 main functional domains (Figure 2A), among which a highly conserved DNA binding domain (DBD) and a less conserved ligand binding domain (LBD). The DBD shares an 82% sequence identity with the human ERα one (83% with human ERβ) and much less with the other NR3 receptors (<62%). The same pattern is observed for the LBD, although this domain is less conserved since it exhibits only 34% amino acid identity with human ERα (35% for human ERβ) and about 20% with other steroid receptors (Figure 2A). The three other domains, namely the A/B region in the N-terminal part, the hinge between DBD and LBD, and the short C-terminal end of the protein, are more divergent, which is a general pattern for NRs [2] (Figure 2A). The recent release of the amphioxus genome confirmed the presence of a single ER gene [35]. In contrast the previously described lamprey ER is more similar to the human ERα with its DBD sharing a 93% sequence identity (93% for human ERβ) and its LBD sharing 55% sequence identity (56% for human ERβ) [8].

### Phylogenetic analysis of ERs

The orthology relationships of the amphioxus and lamprey ER sequences were studied in a phylogenetic analysis of the NR3 family using an exhaustive dataset comprising 69 members of the NR3 subfamily as well as sequences of RXRs as an outgroup. The dataset included the 6 currently known mollusk ER sequences (from *Nucella lapillus, Crassostrea gigas, Marisa cornuarietis, Thais clavigera, Octopus vulgaris, Aplysia californica*), as well as the 2 NR3 sequences previously known from amphioxus (1 ERR [GenBank: AAU88062] and 1 NR3C [JGI: 201600], retrieved from a previous work [36] or from the complete genome sequence [35]). In the resulting phylogenetic tree, the sequence of lamprey ER branches within the ER clade with a high bootstrap support (95%), at the expected position before the split of vertebrate ERα and ERβ (Figure 2B, and for a tree presenting all sequences, see Additional file 1A), as previously shown [8]. The sequence of amphiER branches within the ERs and is located at the base of the vertebrate estrogen receptor group, before the split of ERα and ERβ but after the split of the mollusk ERs (bootstrap value of 81%, Figure 2B). However its precise position within the ER group is poorly supported (bootstrap value of 42%).

Such low bootstrap supports reveal either the weakness of the phylogenetic signal contained in ER proteins, or the presence of two incompatible signals in the data, one supporting the observed position of amphiER within ERs, and the other supporting another position. Whereas the weakness of the signal is not testable, the long branch leading to amphiER in the tree suggests that sites that have undergone a large number of substitutions may account for one of the two signals. Such sites may be saturated to the point that phylogenetic methods are not able to correctly recover their evolution, a situation leading to the long branch attraction artifact [37]. It is therefore important to correctly characterize sites that support the ER position of amphiER: if only fast-evolving sites support this hypothesis, it is probably due to long branch attraction, and an alternative branching should be favored. Alternatively, if slowly-evolving sites support this position, one can confidently identify amphiER as a *bona fide *ER. To characterize sites with respect to their evolutionary rates and the amphiER position they favor, both site likelihoods and site evolutionary rates were computed for all possible positions of amphiER.

**Figure 3:**
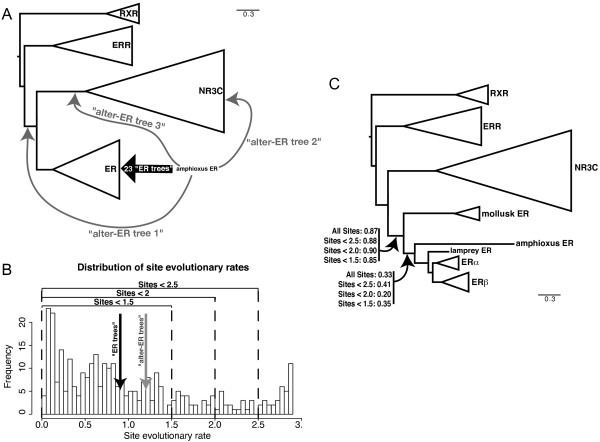
**Phylogenetic analysis of amphiER**. (A) Summary of the 26 best placements of amphiER within a phylogenetic tree comprised of 69 NR3 and 8 RXR sequences. 23/26 topologies ("ER-tree") place amphiER within the ER clade, the 3 remaining topologies ("alter-ER tree") place amphiER either at the base of (ER, NR3C) or within the NR3C family (close to an AR) or at the base of the NR3 family. The mean evolutionary rate of the sites supporting one of the 23 "ER-tree" topologies (0,9) or for the "alter-ER tree" topologies (1,2) are indicated in (B). (B) Distribution of the site relative evolutionary rates. Rates were estimated using an 8 class discretized gamma distribution. The vertical dotted lines correspond to different tentative threshold (2.5, 2, 1.5), above which sites have been discarded due to their high evolutionary rate, before reestimating the phylogeny of the consecutive alignment. (C) Estimation of the minimum of Chi2-based and SH-like supports, available in the aLRT-PHYML software, for the branches defining the monophyly of ERs as well as the position of amphiER. 4 trees were inferred using an alignment on which the fastest evolving sites were removed (no site removed, 34, 53 and 82 sites removed out of 323, with a mean evolutionary rate threshold above 2.5, 2.0 and 1.5, respectively).

First, AmphiER was pruned from the tree shown in Figure 2B, and then re-grafted in all 149 remaining branches. This yielded 149 topologies, for which site likelihoods and site evolutionary rates could be computed using PhyML-aLRT. This allowed us to obtain an evolutionary rate per site averaged over all possible positions of amphiER, and therefore independent from the precise position of amphiER in the tree. Additionally, as likelihoods were computed for each of the 149 positions, these positions could be compared according to the Approximately Unbiased test (AU test, implemented in Consel [38]). Out of all the 149 resulting trees, 26 could not be distinguished with the AU test and had a likelihood significantly better than all the other ones (p-value > 0,05). Of these 26 topologies, all but three place amphiER within the ER clade ("ER" trees). The remaining topologies ("alter-ER" trees) place amphiER either at the base of the NR3C clade (comprising the ARs, PRs, MRs and GRs), within the NR3C or at the base of (ER, NR3C) (Figure 3A). Because site evolutionary rates had been computed, sites having a higher likelihood for the "alter-ER" trees could be compared with sites favoring the "ER" topologies with respect to their evolutionary rates. Interestingly, the sites pleading for the "alter-ER" trees evolve significantly faster than the sites pleading for the "ER" trees (mean evolutionary rates of 1.20 and 0.90, p-value < 10^-5 ^with a Wilcoxon-test or p < 0.001 with a an unpaired t-test). This suggests that the "alter-ER" signal in the alignment is probably due to long branch attraction to the NR3C subtree, which might also be at the origin of the low bootstrap support (42%) for the position of amphiER. Conversely, this suggests that amphiER should be considered as an ER, as the signal at the origin of this position does not seem to be artifactual.

An additional test can be run to further confirm this hypothesis, and consists in reestimating the phylogeny using only slowly-evolving sites. For that purpose, the distribution of expected relative evolutionary rates across sites of the alignment was plotted, as found by phyml-aLRT [39,40] (Figure 3B). Fastest-evolving sites were removed from the dataset based on three different rate thresholds (2.5, 2 or 1.5, Figure 3B and 3C), and trees were reconstructed based on the alignments containing only the remaining slowly-evolving sites. These operations did not impact the monophyly of ERs (Figure 3C) or the statistical support. This shows that the clustering of amphiER with vertebrate ERs does not come from saturated sites, which argues against long branch attraction being at the origin of this position [41]. Accordingly, complementary phylogenetic analyses with different methods (bayesian, Neighbor-joining, parsimony) gave similar results (see Additional file 1). From these studies we conclude that amphiER does indeed belong to the ER subfamily, which is confirmed by the general conservation of the exon-intron structure of amphiER with human ERα, especially at two exon-intron splice sites in the DBD and in the LBD after helix 3 (Figure 2C and short red strokes in Figure 1) [42].

### Chordate ERs, including amphioxus ER and lamprey ER, are able to bind estrogen specific response elements (ERE)

To test whether the lamprey ER and the amphioxus ER are able to bind DNA on specific estrogen response elements (EREs), electrophoresis mobility shift assays were performed using a radiolabeled consensus ERE sequence (see Additional file 2). These experiments show that, like vertebrate ERs, amphiER and lampER are able to bind DNA specifically on a consensus ERE. This binding is specific, since a 100-fold excess of non-specific DNA was not able to compete for binding, whereas a 100-fold excess of cold ERE completely suppressed it (see Additional file 2, compare lanes 15 and 17, as well as lanes 19 and 21). ERs contain two major conserved signatures in the DBD, the P-box (CEGCKA), responsible for the binding specificity to response elements, and the D-box, also involved in the DNA binding specificity of the ER dimers (Figure 2A). The P-box is highly conserved in all known ERs, including amphiER and lamprey ER and is different from other NR3 members. AmphiER and lamprey ER also have a well conserved D-box, amphiER D-box containing just a few conservative mutations, (*e.g. *a mutation of an alanine in glycine, Figure 2A). Since the three characterized mollusk ERs (from *A. californica, O. vulgaris *and *Thais clavigera *[11,12,24]) also bind EREs and since the P-box and D-box are well conserved in all known ERs, including those from mollusks, ERE binding appears to be a feature specific to all ERs.

### Lamprey ER, but not amphioxus ER, is able to induce transactivation of a reporter gene in response to estradiol stimulation

**Figure 4:**
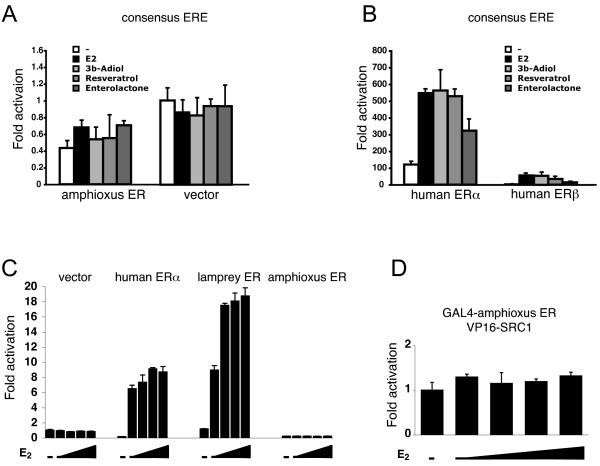
**LampER is activated by E_2 _whereas amphiER is not activated by ER agonists**. The pSG5 constructs containing either amphiER (A), human ERα or human ERβ (B), were tested in transfected Cos 7 cells for their ability to activate the co-transfected cognate ERE-luc reporter plasmid after E_2 _stimulation (10^-6^M). (C) GAL4-LBD constructs from several chordate ERs were tested in transfectec 293 cells for their ability to activate a (17m)5x-G-luc reporter plasmid in the presence of increasing doses of E_2 _(10^-9^M to 10^-6^M). (D) Mammalian two-hybrid SRC1 recruitment assay. The GAL4-amphiER-LBD chimera was used with the coactivator SRC1 fused to the strong activation domain VP16 to transfect 293 cells in the presence of increasing doses of E_2 _(10^-9^M to 10^-6^M).

The transactivation ability of lamprey ER and amphiER was then compared with that of human ERα. AmphiER failed to induce transcription of a reporter construct containing a consensus ERE in front of a minimal promoter in transfected mammalian cells after stimulation by the natural vertebrate ER ligand, estradiol (E_2_) as well as a wide variety of other vertebrate ER ligands (the natural agonist 3β-androstenediol [43], and the phytoestrogens resveratrol [44] and enterolactone [45]) (Figure 4A and 4B). In order to improve the detection sensitivity, was also tested the transactivation capacity of amphiER in response to E_2 _as a construct containing only the LBD fused to the GAL4 DNA-binding domain. In this case again, no activation was detected (Figure 4C). In agreement with this result, no recruitment of the coactivator SRC1 (an homologue of which is present in the amphioxus genome, see Discussion) was detected in mammalian two-hybrid assay (Figure 4D). However lampER is activated by E_2_, with an intensity comparable to humanERα (Figure 4C), which suggests that the lamprey ER is a high affinity E_2_-dependant transcription factor.

**Figure 5:**
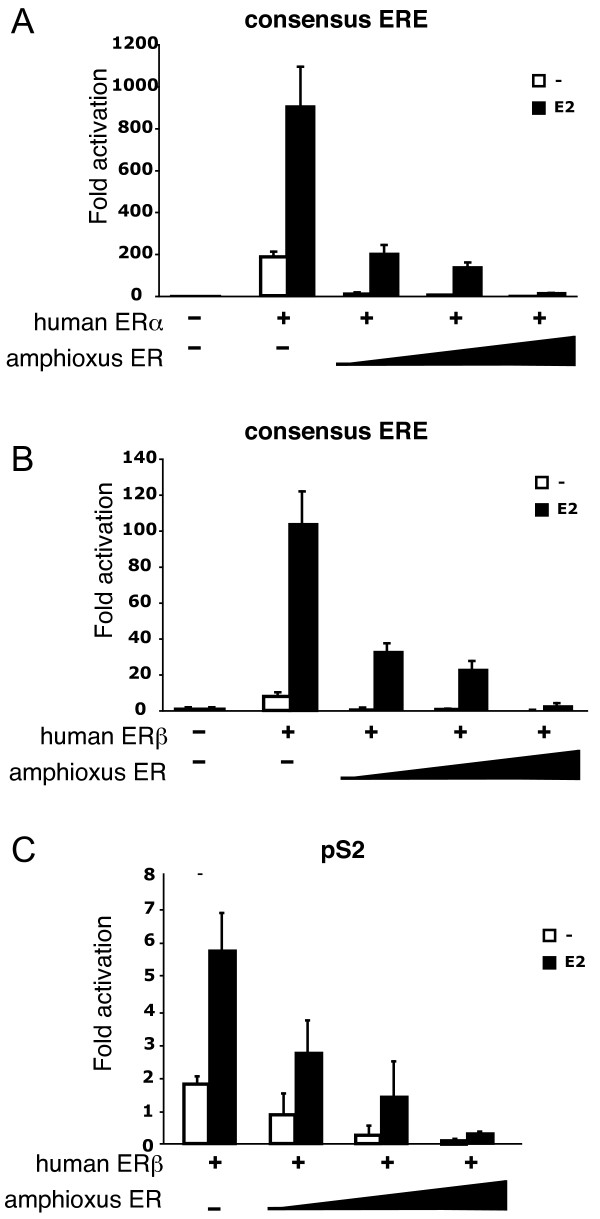
**The amphioxus ER acts as a dominant negative estrogen receptor**. A pSG5 construct containing human ERα (A) or human ERβ (B) was tested in transfected HeLa cells for its ability to activate the co-transfected cognate ERE-luc reporter plasmid after E_2 _stimulation (10^-6^M) in presence of increasing doses of the amphiER construct. (C) A pSG5 construct containing human ERβ was tested in transfected HeLa cells for its ability to activate the co-transfected ps2 promoter after E_2 _stimulation (10^-6^M) in the presence of increasing doses of the amphiER construct.

Since amphiER is able to bind DNA but is unable to activate transcription of a reporter gene, the dominant negative capacity of the amphioxus protein was tested. A dose-dependent decrease in the reporter gene activity was clearly visible in 2 different cell lines when increasing amounts of the amphiER plasmid were added together with constant amounts of human ERα or ERβ in transient transfection experiments. This decrease was observed both with synthetic consensus EREs (Figures 5A and 5B. See also Additional file 3) and with the natural ERE present in the classical ER pS2 target gene (Figure 5C). Apparently, amphiER is able to compete with human ERα or ERβ for binding to the ERE sites present in the reporter constructs, and in doing so, prevents ERα and ERβ from inducing transcription, which results in a decrease in reporter gene activity. Thus, in contrast to *Aplysia, Octopus *or *Thais *ER [11,12,24], amphiER does not display constitutive transcriptional activity under our experimental conditions and rather exhibits an inhibitory effect (Figure 4). This clearly shows that the absence of transcriptional activity observed here is not an artifact linked to a poor expression of the construct but rather reflects the inability of amphiER to activate transcription in mammalian cells.

### LampER is an estradiol receptor whereas amphiER is not able to bind ER ligands except the synthetic compound Bisphenol A

**Figure 6:**
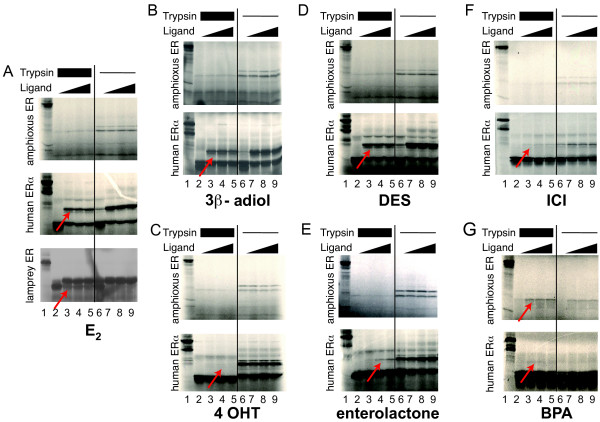
**Limited proteolysis of lampER with E_2 _and of amphiER with various ER ligands**. Human ERα was used as a positive control. lane 1: undigested protein, lanes 2–5, 6–9: digested protein in the absence (lane 2 and 6) or presence (lanes 3–5 and 7–9) of ligand (10^-3^M to 10^-5^M). 2 different trypsine doses are shown, indicated by thick or thin bars above each panel. The ligands are (A) estradiol, (B) 3β-Androstane-diol, (C) 4-hydroxytamoxifen, (D) diethylstibestrol, (E) enterolactone, (F)ICI-182780 and (G) bisphenol A.

In order to confirm that lamprey ER is an E_2 _receptor and to better understand the molecular basis behind the inability of the amphiER to become transcriptionally activated by estradiol stimulation, E_2 _binding by lamprey ER and amphiER was tested *in vitro*. For that purpose, limited proteolysis assay allows to assess whether addition of different putative ligands can induce a conformational change in amphiER [13]. Using this method the ligand induced conformational change of the LBD is revealed by the alteration of the receptor sensitivity toward proteolytic digestion by trypsin. As expected, E_2 _was able to protect human ERα from proteolysis (Figure 6A). Interestingly lamprey ER was also protected from proteolysis by E_2 _even at the lowest concentration tested (Figure 6A), thus confirming the results of the transactivation assays that the lamprey ER is a high affinity E_2 _receptor. In contrast no protection of amphiER by estradiol was observed, even at very high ligand concentrations (10^-3^M) (Figure 6A). Since estradiol does not protect amphiER from proteolysis, several other classical ER ligands were tested, such as the synthetic ER agonists diethylstilbestrol [46], 4-hydroxy-tamoxifene [46] or bisphenol A (BPA) [47], the natural agonist 3β-androstenediol [43], the phytoestrogen enterolactone [45] or the synthetic ER antagonist ICI-182780 [46]. All compounds were able to bind to human ERα (Figure 6B to 6G) as expected, whereas none but BPA was able to bind to amphiER (Figure 6G). However, BPA did not induce transactivation by amphiER in mammalian cells reporter assay, and did not induce recruitment of the coactivator SRC1 either (see Additional file 4).

In order to rule out the possibility that amphiER is activated by a compound related to E_2_, a large panel of 14 other steroids and cholesterol derivatives were tested for their ability to bind and activate amphiER. None of the tested compounds, even at high doses, had any effect on amphiER transcription activity (see Additional file 5A). Accordingly, no recruitment of the coactivator SRC1 by amphiER was detected in mammalian two hybrid assays (See Additional file 5B). The most probable explanation is thus the lack of binding by those compounds to amphiER (See Additional file 6).

Taken together these results show that the ER from lamprey behaves as a "classical" ER since it binds DNA on a classical ERE and is activated by binding E_2_. On the other hand, though the single ER from amphioxus is able to bind the ERE, it does not bind any tested ER ligand and cholesterol derivative, except bisphenol A. However, no transcriptional activity was detected upon stimulation by any of the tested ligands. Since none of the mollusk ERs sequenced up to now binds E_2 _either, our data suggest that E_2_-binding by ER is restricted to vertebrates, implying that vertebrates specifically gained the ability to be regulated by E_2 _(see Discussion).

### Ancestral reconstruction of steroid receptors

**Figure 7:**
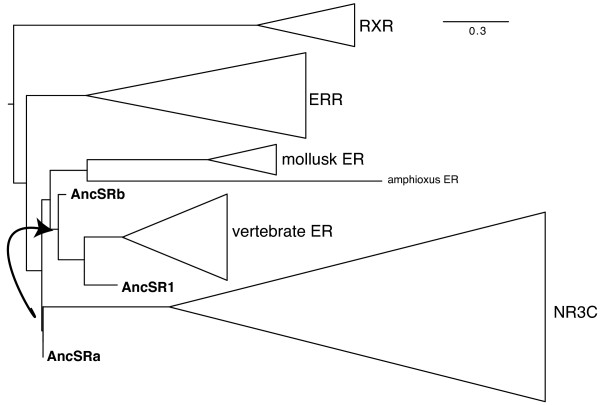
**The reconstruction of the ancestral sequence of steroid receptors is sensitive to taxonomic sampling**. The ancestral sequence of ER and NR3C was inferred using either a complete dataset (AncSRa) or a partial dataset (AncSRb) where 5 mollusk ER sequences as well as amphiER and amphiNR3C were omitted. The position of those sequences within the phylogenetic tree calculated with the complete dataset was compared. The position of a previously described ancestor (AncSR1) is indicated as well. Triangles represent the different NR clades. For the complete tree, presenting all the 80 sequences present in the tree, see Additional file 7.

In previous analyses that discussed the evolution of ERs, it was argued that estradiol binding was an ancient function of all sex steroid receptors (SRs, comprised of ERs and NR3C members) and that the binding to other steroids was more recent, with estradiol binding ability getting restricted to ERs [8,11]. Those conclusions were based on the reconstruction of the ancestral SR sequence [11]. Since the finding that estradiol binding is not shared by all ERs but restricted to vertebrate ERs contradicts this hypothesis, and to get better insight into this apparent contradiction, the sequence of the ancestral steroid receptor was reestimated. When the ancestor (AncSR1) was first "resurrected", only one non-vertebrate sequence was available [8,11]. The impact of more non-vertebrate sequences (including amphiER) was thus tested on the reconstruction of the ancestor of steroid receptors. The sequence of the ancestral steroid receptor (AncSRa), at the node grouping ERs and NR3C, was inferred using PAML 4 [48], from the alignment described previously (the study was restricted to DBD and LBD) and the topology shown in Figure 2B. The predicted sequence resembles AncSR1 (Figure 1) with 12 out of 18 amino acids involved in ligand binding [49] being ER-like (Figure 1). However, important differences were noticed between AncSR1 and AncSRa. First one of the 3 amino acids making direct contacts with E_2 _is different in AncSR1 and AncSRa: at this position, AncSR1 is vertebrate-ER like (a His residue is present at position 524 of humanERα, located in helix H10–H11, in green in the alignment, Figure 1) whereas the amino acid is different in AncSRa and is mollusk-ER like (Tyr instead of His) and mutations at this site have been shown to impair ERα activity in human [50,51]. Second, when a phylogenetic tree is built including both ancestral sequences AncSR1 and AncSRa as well as various NR3 sequences, AncSRa branches deep in steroid receptors as expected since it was built on the same dataset (Figure 7. For a complete tree presenting all leaves, see Additional file 7) but AncSR1 branches close to the vertebrate ERs, which is surprising. In order to determine whether the taxonomic sampling, and not details of the alignment, was responsible for these differences, we calculated a second SR ancestral sequence (AncSRb) using a smaller dataset: taxon sampling was reduced by removing most of the non-vertebrate steroid receptor sequences from the alignment (the amphioxus ER, the amphioxus NR3C as well as 5 out of the 6 mollusk ER sequences were removed to obtain a dataset closer to the one used in [8]). In this case, AncSRb branches next to AncSR1, closer to the ER clade than to AncSRa (Figure 7). This result clearly shows that the reconstruction of ancestral sequences is influenced by the set of sequences available and that restricted taxonomic sampling biases ancestral SR sequences towards vertebrate ER sequences. Therefore conclusions based on such an analysis (specifically that the ancestral ER was able to bind estradiol) should be considered as only tentative, since taxonomic sampling of available steroid sequences is very much vertebrate-centered. Overall, taken together, our data do no support the hypothesis that the ancestral steroid receptor was an estradiol receptor.

## Discussion

### The amphioxus ER does not bind estradiol

In this paper we cloned and functionally characterized the lamprey and amphioxus orthologues of the human estrogen receptors. Our results show that lampER binds estradiol whereas amphiER does not. We propose that 3 types of ERs can be distinguished, depending on their ligand binding properties: vertebrate ERs (including lamprey) are the only *bona fide *estradiol receptors, mollusk ERs do not bind estradiol and are constitutively active transcription factors and amphiER does not bind estradiol and is transcriptionally silent in mammalian cells. This is supported by two points: (i) the experimental approach developed here is biologically relevant since the binding of bisphenol A (BPA) to amphiER was observed using the same experimental conditions as for E_2 _suggesting that amphiER is correctly folded and that a ligand binding pocket is likely to be present. (ii) One of the three key amino acid positions within the LBP of amphiER (Cys 531, located between the helixes H5 and H6, in green in Figure 1) diverges from vertebrate ERs (Arg 394 in human ERα), whilst the two other key positions (Glu 490 and His 659 in amphiER, located in helix H3 and H10–H11 respectively, in green in Figure 1) are conserved with the vertebrate ERs (amino acids corresponding to Glu 353 and His 524 in human ERα), suggesting that potential contacts between amphiER and estradiol are impaired. Accordingly, a recent *in silico *study of amphiER ligand binding ability confirmed an "unusual ligand recognition in amphioxus ER" [52].

It was unexpected that no effect of the synthetic ER agonist BPA was detected in the transactivation assay of the receptor in mammalian cells since BPA induces a conformational change of amphiER. This apparent absence of coactivator recruitment (see Additional file 4B) resulting in no transcriptional activity in response to BPA can be interpreted in several ways: (i) because of the different geometry of the ligand-binding pocket in amphiER, BPA behaves as an antiestrogen (partial agonist or even a partial antagonist) and blocks the transcriptional activation properties of amphiER, for instance by inducing a conformational change that does not allow coactivator recruitment (like human ERα and 4-raloxifen, [53]) or by excluding amphiER from nucleus (like ICI-182,780 with human ERα, [54]). (ii) Alternatively, the coactivator interface of amphiER does not fit with mammalian coactivators, resulting in artifactual loss of activation. However, the conservation of the amino acids involved in co-activator interaction, compared to human ERα does not support this hypothesis (sites indicated with a star in Figure 1, as described in [55]). Among the divergent sites, at a position implicated in the charge clamp necessary for coactivator contact, amphiER contains an aspartate (D677) instead of a glutamate in humanERα (E542). Importantly, the divergence (E->D) is conservative and preserves the negative charge of the amino acid, which is important for interaction with the lysine from helix 3 (conserved in amphiER) to form this charge clamp [56]. In addition, a unique orthologue of the p160 family of coactivators was found in the amphioxus genome [35] and its overall conservation with its 3 human ohnologues (genes that have been duplicated during the two rounds of whole genome duplications in the chordate lineage [57]) is good. (iii) Interaction between mammalian chaperones like HSP90 and amphiER is impaired, leading to improper binding to the hormone [58]. Taking these results into account, it will be interesting to test the effect of BPA on the subcellular localization of amphiER and to study if other related compounds are able to bind and/or activate amphiER. In addition, it will be important, when cell cultures from amphioxus are available, to check the activity of amphiER in a monospecific transient transfection assay. It should be remembered that some orphan receptors such as ERRs are thought to have no natural ligands even if they are able to bind synthetic compounds [59]. More generally, the precise status of amphiER in terms of ligand binding remains an open question. It is nevertheless clear, and this is an important issue for the current evolutionary debate, that amphiER is not able to bind estradiol.

### Is there any receptor for estradiol in amphioxus?

The observation that amphiER does not bind E_2 _is indeed a surprising observation since E_2 _was detected in amphioxus by RIA, the hormonal production being correlated with breeding season [15]. Several aspects of steroid metabolism were described in amphioxus [60] and the homologues of many enzymes necessary for estradiol synthesis in mammals were cloned from amphioxus ovaries [15,61]. Of particular interest is the report of an aromatase gene (CYP19) in amphioxus, which suggests that the crucial step in estradiol synthesis is indeed possible in amphioxus. These experimental data were recently confirmed by the analysis of the complete amphioxus genome sequence [35]. It may be that, in amphioxus, the active sex hormone is an E_2_-derivative [62] or another sex hormone, like in the case of androgens in lamprey [63], and this derivative is still to be discovered. In a similar way, we recently demonstrated that the amphioxus TR orthologue does not bind T_3 _or T_4_, the classical thyroid hormones, but deaminated derivatives TRIAC and TETRAC, which are able to induce amphioxus metamorphosis [64].

A second possibility is that E_2 _itself has a central role in sex maturation in amphioxus, and that the functional estrogen receptor in amphioxus is different from amphiER. Several candidates are possible. First, there is another steroid receptor in amphioxus (amphiNR3C in Figures 1 and 2) [35] that exhibits several ER-like features. Its P- and D-boxes are closer to ERs than to vertebrate NR3C (Figure 2A). The sequence identity of its LBD with human ERα (37%) and with NR3C members (35%) are similar. Moreover, most of the amino acids involved in ligand binding are more ER-like than AR-, PR- or MR-like (Figure 1). However it is the only NR3C receptor (orthologous to AR, PR, MR and GR) found in the amphioxus genome. Thus if amphiNR3C plays the role of an estradiol receptor, this suggests an absence of a "classical" steroid receptor able to bind testosterone, progesterone or corticoids. Alternatively, a non-nuclear receptor could mediate E_2 _action in amphioxus. Indeed, several non-genomic effects of estradiol were reported in mammals involving GPCRs (for reviews see [65-67]). For instance, very recently, a high affinity receptor for the steroid androstenedione linked to the membrane, was described in lamprey [68] and a GPCR with high affinity for progestines was isolated from sea trout [69].

### Implications for the evolution of ERs

**Figure 8:**
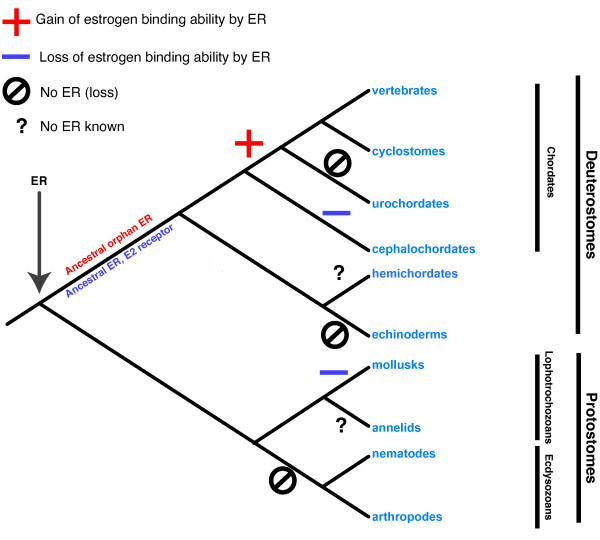
**Model of evolution of the ligand binding ability of ERs**. On a classical phylogenetic tree of bilaterians, data available on the binding ability of all known ERs have been indicated. Two hypotheses are compared in terms of parsimony, whether the ancestral ER was liganded (in blue) or not (*ie *an orphan receptor) (in red). This result displays different costs in terms of parsimony: one unique event of gain specifically in vertebrate for the "ancestral ER orphan" hypothesis against at least two parallel events of loss of binding for the "ancestral ER binding E_2_" hypothesis. In addition, three events of loss of the ER gene in urochordates, echinoderms and insects+nematodes are implied by the current distribution of the gene across metazoans.

The absence of E_2 _binding by the amphioxus estrogen receptor has interesting consequences for the evolution of SRs and ERs. Indeed, only the well characterized gnathostome ERs and the lamprey ER (studied here) have been shown to mediate E_2 _action. Outside vertebrates, all the ERs studied so far (in mollusks and amphioxus) do not bind E_2 _[11,12,24-26]. Parsimony implies that the function of estradiol in the bilaterian ancestor was not mediated by ER and that ER had another function. Only later during evolution, in the vertebrate lineage, ER would then have gained the ability to be activated by E_2 _and to mediate the hormonal action of this compound (Figure 8). The alternative scenario (ancestral E_2 _binding and independent loss of either ER itself or E_2 _binding to ER in mollusks and invertebrate deuterostomes) is more costly in terms of evolutionary events, even if the hypothesis of an NR3C orthologue binding E_2 _is taken into account. Thus, taken together, our results do not support previous scenarios of steroid receptor evolution based on a reconstruction of the ancestral steroid hormone receptor AncSR1 [8,11].

To describe the evolution of a protein, being able to study ancestral sequences at different nodes of a phylogeny would obviously provide historically relevant information that is not available otherwise [70]. However such sequences have disappeared long ago and can only be statistically estimated. The accuracy and bias of these estimations therefore need to be investigated. Indeed, functional studies of ancestral sequences are of any value only if the ancestral reconstruction is reliable enough. The confidence associated with the previously published ancestral steroid receptor is quite low. Indeed, the overall accuracy of the reconstruction of the LBD (AncSR1) was only 62% [11]. This is similar for the ancestor inferred here (AncSRa) on an enriched dataset, with an overall accuracy of the DBD+LBD of 70%. Moreover, amino acid uncertainty was high at many sites of AncSRa and AncSR1: more than 60 sites have more than 1 possible amino acid with a probability superior to 0.2. If one were to make an exhaustive study, one would need to reconstruct and test more than 10^24 ^potential proteins (if all possible combinations of amino acids with probability > 0.2 were tested). In fact several of the sites involved in ligand binding have low probabilities. Examples of more reliable reconstructions of nuclear receptors have been published, *e.g. *the ancestor of MR and GR (mineralocortoid and glucocorticoid receptors) in which the overall accuracy of the LBD was above 99%, with no disrupting mutation at any site [71]. The reconstructed ancestor of RARs also showed a high average confidence (99% [13]). This discrepancy between results obtained on the ancestor of all steroid receptors or merely of MR and GR for instance, can be explained by the higher sequence divergence observed among all SRs than simply among subfamilies MR and GR (see branch length in Figure 2B). Consequently, the uncertainty associated to the sequence of the ancestral steroid receptor as estimated with nowadays methods is probably too high to provide a firm basis for evolutionary conclusions. Moreover, the phylogenetic reconstruction of ancestral sequences has been shown to be biased towards the most frequent (and more stable) amino acids, resulting in an under-estimation of the less frequent amino acids (the stability of the ancestral protein is then over-estimated [72]). In reconstructions of ancient proteins, where the evolutionary signal has been lost due to a high number of substitutions, such biases might be problematic. Thus, current reconstruction methods do not seem powerful enough to infer a biologically meaningful ancestral steroid receptor given the amount of divergence between sequences.

Nonetheless, all these reservations put aside, it is surprising that the previously reconstructed ancestral SR, is vertebrate ER-like. As almost all the extant sequences used as matrix for the reconstruction came from vertebrates and led to the estimation of a "vertebrate-like" ancestral sequence, the same ancestral steroid receptor as previously published [11] was estimated, but adding more sequences from various taxa. This reconstruction was done using a phylogeny equivalent to the one previously published [11]. Using this approach, the new AncSRa is more divergent from vertebrate ERs than AncSR1. Interestingly AncSR1 was shown to bind E_2 _with a very low affinity (250 times lower affinity than human ERα [11]), suggesting that AncSRa may be an even worse estradiol receptor.

The bias of AncSR1 towards vertebrate ERs is explained by a lack of non-vertebrate sequences used for the reconstruction. Indeed, removing some non-vertebrate sequences from our dataset leads to an estimation of an ancestral steroid receptor that is more "vertebrate ER"-like (AncSRb in Figure 7). The clustering of AncSRb with the ER clade and the exclusion of AncSRa from the ER clade were supported with good statistical values (minimum of Chi2-based and SH-like supports of 0.83 and 0.89, respectively). Those data show that AncSR1 reconstruction was probably sensitive to the vertebrate bias in the data set (Figure 7). Overall, we suggest that analysis based on ancestral reconstructions should be taken as tentative, especially in case of low statistical confidence and limited taxonomic sampling. In case of the ancestral steroid receptor, even if exhaustive taxonomic sampling is necessary, phylogenetic signal is weak and the resulting confidence is quite low. Thus we think that even if the ancestral sequence built here is biologically more relevant than previously calculated ones (because of better taxonomic sampling), it remains quite uncertain. Consequently conclusions regarding the ancestral steroid receptor should be based mostly on comparative characterization of extant receptors. In that case, all the data based on invertebrate ER receptors (from mollusks and amphioxus) support an ancestor of steroid receptors that was not able to bind estradiol. This conclusion will obviously require the functional characterization of ERs from other protostome phyla in order to carefully check if this observation is general. Thus, available data converge towards a re-evaluation of the ancestral status of estrogen receptors.

### Sequence conservation reflects functional constraints: ligand binding ability is more recent than DNA-binding ability

From our and previous studies, only vertebrate ERs are able to bind and activate transcription under estradiol stimulation [11,12,24-26]. The LBD of amphiER is more divergent from its vertebrate counterparts (ca. 34% amino acid identities) than the LBD of other liganded amphioxus nuclear receptors such as amphiRAR (ca. 58%), which has been shown to bind the same ligand as its vertebrate homologue [7,13]. This suggests that a conserved functional feature (e.g. binding to the same ligand) is reflected in the sequence conservation of the LBD.

The same observation can be done concerning the DBD since all ERs, including amphiER, have a highly conserved DBD and are able to bind EREs. Thus, for this domain also, a conservation of the function is reflected in sequence conservation.

Accordingly with this notion, the LBD of invertebrate ERs is highly divergent but their DNA binding domain, as well as other functionally important domains not directly linked to ligand binding such as the dimerization interface, or the amino acids responsible for interaction with the co-activators [55] are well conserved. This is true for amphiER as well as mollusk ERs. This strongly suggests that amphiER is a *bona fide *NR regulating ERE-containing genes in an E_2_-independent manner. Post-translational modifications such as phosphorylation or the presence/absence of other receptor-interacting proteins such as transcriptional coactivators have been shown to regulate unliganded nuclear receptors [73]. Whether one of these mechanisms acts to regulate the activity of invertebrate ERs or if those receptors have unknown ligands still to be identified remains to be explored. Anyway our observations strongly suggest that for ERs, the DNA binding function of the receptor as well as its interaction with co-regulators have been conserved due to selective pressure. Interestingly, when studying the AncSRa, the P- and D-boxes in the DBD are ER/ERR-like (Figure 1), suggesting that ER/ERR DNA binding ability is ancestral, in accordance with the fact that these are the only receptors of the NR3 family found in invertebrates. This difference in the selection pressure between DBD and LBD has been proposed to be a general evolutionary pattern for the whole NR family [7]. The plasticity of the ligand binding ability of NRs was recently illustrated in the case of RXR-USP where the ability of the receptor to be regulated by a ligand was suggested to have been subject to several successive episodes of gain and loss during evolution [74].

### Evolution of endocrine systems: refinement of the ligand exploitation model

The ligand exploitation model hypothesizes how new hormones and new receptors appear during evolution. It suggests that the ancestral ligand is the last metabolite of a synthesis pathway [8]. According to this model, the ancestral steroid ligand was estradiol (and the ancestral SR bound estradiol). During evolution, other steroid receptors appeared by duplication of the ancestral ER and gained the ability to bind other steroids, intermediate in the synthesis pathway (like testosterone or progesterone).

Our findings on the evolution of ERs do not support the ligand exploitation model, since our data strongly suggest that the ancestral ER did not bind estradiol. However, as estradiol has been detected in deuterostomes as well as protostomes (for instance in vertebrates, amphioxus, echinoderms, mollusks, for review, see [75]), steroid signaling may have been already present in bilaterian ancestor. However, up to now, the ancestral steroid molecule remains to be determined. If estradiol is an ancient hormone, it then probably bound another receptor and later on ER gained the ability to recognize it, as did other steroid receptors for their extant ligand. Thus the evolution of steroid system intermingles two distinct processes, the evolution of the receptor on one hand, and the evolution of the ligand on the other.

The receptor can evolve by point mutations and change its affinity for a ligand towards another. This idea was convincingly exemplified in the case of corticoid receptors (the ancestor of MR and GR) for which it was recently demonstrated that ability of the ancestral vertebrate corticoid receptor to bind gnathostome-specific hormone aldosterone (a MR ligand) was a by-product of its ability to bind the ancestral ligand 11-deoxycorticosterone (DOC) [71]. GR gained the ability to bind cortisol only in the gnathostome lineage, in parallel to endogenous synthesis of the hormone [16,71]. This detailed study shows that a receptor binding a given ligand can acquire affinity for compounds present in the cell that are structurally close to its natural ligand: this refines the ligand exploitation model, since new ligands are not necessarily precursors of ancient ligands, simply compounds present in the cell and structurally close to the ancestral ligand. Similar conclusions were drawn previously in the case of RAR evolution [13]. It has to be emphasized that the pool of available compounds is also subject to evolutionary changes in parallel. For instance, the spatiotemporal production of estradiol is variable in the different vertebrate groups (reviewed in [76]). Glucocorticoids differ in mouse (cortisol) and in human (corticosterone), with both hormones being GR ligands [77]. There are several androgens in teleost fishes, with 11-ketotestosterone being teleost-specific [78]. As there are 2 androgen receptors (ARs) in teleost fishes, from a whole genome duplication [79], the study of the ligand-binding ability of those ARs is a potentially interesting case for the evolution of endocrine systems. As highlighted by Bridgham et al. (2006), lamprey does not produce cortisol [71]. In accordance, their genomes do not contain the sequence corresponding to the enzyme responsible for cortisol synthesis (11b-hydroxylase) and in general classical steroids except estradiol are rarely found in lamprey. This suggests that the steroids actually found in lamprey are different from the ones found in mammals (reviewed in [80]). Those cases exemplify the largely underestimated diversity of endocrine systems: except for lamprey and some teleost fishes, the hormonal pool of animals remains largely unknown. As proposed for the study of steroid receptors, a comparative approach should be applied to determine the metabolism of steroids in poorly studied animals. Indeed, the hormonal pool of such animals is usually evaluated from the presence/absence of putative orthologues of mammalian enzymes. As the enzymatic machinery involved in hormonal metabolism has a very labile activity (reviewed in [81,82]), equating orthology with functional identity might be unreliable.

The evolution of steroid receptors can be replaced in the more general context of ligand-nuclear receptor co-evolution. The evolution of the NR1H subfamily, that includes receptors for other steroidal compounds, like the major transcriptional regulator of bile salt synthesis farnesoid × receptor (FXR), the pregnane × receptor (PXR), the vitamin D receptor (VDR) or liver × receptor (LXR)/ecdysone receptor (Ecr), has been extensively studied and is not in line with the ligand exploitation model [83-85]. For instance, comparative functional studies of FXRs from various chordate species showed that the vertebrate FXRs bind "late" cholesterol derivatives (from a complex synthesis pathway) but are thought to have evolved from an ancestral FXR that bound early cholesterol derivatives (from a simpler synthesis pathway) [83].

In other cases, the evolution of ligand binding is more "chaotic" with close orthologs having a selective ligand binding ability that varies extensively (vertebrate VDRs are very well conserved when PXRs have the widest ligand repertoire of all NRs) [83].

These complex histories are probably linked to specific function of some of those NRs, considered as xenotoxic compounds "sensors". This tight relationship with the unstable environment probably makes receptors like FXR and especially PXR more prone to fast evolution [86]. Yet they illustrate the impressive variety of scenarios of NR evolution.

## Conclusion

In this article, we demonstrated that vertebrate ERs (including lamprey ER) are estradiol receptors whilst non-vertebrate ER (including amphioxus ER) are not. The most parsimonous scenario proposes that the ancestral ER was not able to bind estradiol and that it had another function. It later gained the ability to be regulated by estradiol, specifically in the vertebrate lineage. However, additional critical data remains to be discovered in poorly studied taxa [62]. To fully understand the evolution of steroid signaling pathway, a larger number of taxa need to be targeted for detailed comparative studies. More precisely, ERs and other steroid receptors should be cloned from widely distributed taxa, especially in protostomes. Enzymes involved in steroidogenesis should also be cloned and characterized, to understand the evolution of steroid availability. In order to avoid the blinders of a "vertebrate-centered" view, it is of particular importance to establish the steroid hormone repertoire of an enlarged animal panel, including more protostomes. The description of various endocrine systems will certainly be relevant to the early evolution of hormone signaling.

## Methods

### Cloning of amphiER

An initial piece of amphiER was obtained by degenerate PCR on different RT reactions from total RNA extracted either from developing B. floridae embryos and larvae (at 13 h–15 h, 28 h, 36 h, 48 h or 3 d–4 d of development) or from B. floridae adults. The oligonucleotides used were as follows: forward primer 5'-TGYGARGGITGYAARGCITTYTT-3' and reverse primer 5'-GTRCAYTSRTTIGTIGCIGGRCA-3'.

The touchdown PCR program used was as follows:

5' 94 degrees

5× (30" 94 degrees, 1' 55 degrees, 1' 72 degrees)

5× the same cycle, but at 50 degrees annealing temperature

5× the same cycle, but at 45 degrees annealing temperature

5× the same cycle, but at 40 degrees annealing temperature

25× the same cycle, but at 37 degrees annealing temperature

7' 72 degrees

All degenerate PCRs irrespective of the RT reaction template used yielded a 83 bp fragment of amphiER. The fragment was sequenced on both strands and used for the design of oligonucleotides for 5' and 3' RACE experiments with the Invitrogen GeneRacer Kit. The template for the RACE experiments was pooled total RNA from 13 h–15 h B. floridae embryos and from B. floridae adults. In addition to the oligonucleotides provided by the kit, for the 3' RACE, the following primers were used:

3' RACE, 1st PCR: 5'-AACGGAGCATTCAGCAAGGTC-3'

3' RACE, 2nd PCR: 5'-GCATTCAGCAAGGTCAGACAG-3'

5' RACE, 1st strand cDNA synthesis: 5'-ATGTAATCTGTCTGACCTTGC-3'

5' RACE, 1st PCR: 5'-CTGTCTGACCTTGCTGAATGC-3'

5' RACE, 2nd PCR: 5'-TCTGACCTTGCTGAATGCTCC-3'

The protocols for the 1st and 2nd round of PCR experiments are given in the Invitrogen GeneRacer Kit. The 3' and 5' RACE products were subsequently sequenced on both strands and used for the design of oligonucleotides for the full-length cloning of amphiER: forward primer 5'-CGGCGAAGCGAAGAAGATCGAG-3' and reverse primer 5'-CTTAACCGATACTAACGGAACAG-3'. The full-length amphiER was obtained by PCR on pooled RT reactions from total RNA extracted from B. floridae 13 h–15 h embryos, 3 d–4 d larvae and B. floridae adults. The PCR protocol used was as follows:

10' 94 degrees

5× (30" 94 degrees, 30" 55 degrees, 2' 72 degrees)

35× the same cycle, but at 50 degrees annealing temperature

10' 72 degrees

The full-length amphiER clone resulting from this PCR is 2279 bp long, was cloned into the pCR2.1 vector (Invitrogen) and subsequently sequenced on both strands.

### Plasmid constructs and reagents

Full length amphiER were amplified by polymerase chain reaction (PCR) and the obtained fragments were inserted into a pSG5 vector between EcoR1 sites. Lamprey ER was a generous gift from JW Thornton. Human pSG5-ERα and pSG5-ERβ and the 3xERE-Luc luciferase reporter construct have been described previously [87]. The pS2-Luc reporter construct encompasses an 1100 bp estrogen-responsive region of the human pS2 promoter inserted into the pGL3 basic vector (Promega). Chimeras comprising the GAL4 DNA-binding domain fused with the LBD of the human ERα (residues 251 to 595), the LBD of amphiER (residues 364 to 705), the LBD of lampER (residues 234 to 554) have been cloned in the pG4MpolyII vector. 17β-estradiol, genistein, 3β-androstenediol, resveratrol, cholesterol, cholic acid, chenodeoxycholic acid, 22^®^-hydroxycholesterol, 20-Hydroxyecdysone, pregnenolone, trans-Dehydroandrosterone (DHEA), corticosterone, progesterone, 4-androstene-3,17-dione, estrone, testosterone, 5α-androstan-17β-ol-3-one and 1a,25-Dihydroxyvitamin D3 (calcitriol) were purchased from Sigma. Enterolactone was a generous gift from Dr Sari Mäkelä [88].

### Phylogenetic analysis of NR3

Protein sequences of NR3 family members were obtained from GenBank by BLAST search using *Homo sapiens *ERα as a query. Eight additional sequences from the closely related RXR group were also obtained to serve as outgroup sequences. For accession numbers of the sequences used, see Additional file 8.

The retrieved sequences were aligned using the muscle 3.6 program [89] and the resulting alignment was manually corrected with SEAVIEW [90]. Phylogenetic tree was calculated by maximum likelihood as implemented in PhyML version 2.4.3 under a JTT substitution matrix plus a eight-category gamma rate correction (α estimated) and with the proportion of invariant sites estimated. Both the DBD and the LBD were used. Robustness was assessed by bootstrap analysis (1,000 repetitions) [91].

The Bayesian inference was done using the program MrBayes 3.1.2 [92]. Two simultaneous independent runs were performed. For each run, one chain was sampled every 100 generations for 1,000,000 generations after the burn-in cycles, until the average SD of split frequencies was <0.01; additionally, the potential scale reduction factors of the parameters were close to or equal to 1, which indicates that the runs had most probably converged. The neighbour-joining (Poisson correction) and maximum parsimony trees were done with Phylo_win [90].

### Likelihood-based tests of alternative topologies placing amphiER at all possible positions in the tree

The 149 trees were built by reconnecting amphiER from the maximum likelihood tree, into the 149 possible positions. The branch length and the different parameters of the obtained trees were re-estimated using PhyML. Likelihood-based tests of the 149 alternative topologies were calculated using CONSEL: site-wise log-likelihood values, available as output of PhyML, were used to calculate the P-values of the different positions according to the AU test with the software R.

### Ancestral sequence reconstruction

The aminoacid sequence of the ancestral AncSRa and AncSRb was inferred only for the most conserved part of the alignment, *i.e. *the DBD and LBD (defined as in Figure 1). The ancestral sequences were reconstructed by maximum likelihood as implemented in PAML [48], under the JTT substitution model and a gamma distribution with 8 categories of rates across sites, using the tree described in Additional file 1A for AncSRa and a the same topology truncated of the mollusk ER sequences and the amphioxus NR3 sequences, after reestimation of the branch lengths using phyml (JTT+γ) for AncSRb.

### Electrophoretic Mobility Shift Assay (EMSA)

EMSAs were performed as previously described [93]. Where indicated, a 10- and 100-fold molar excess of 30-bp unlabeled oligonucleotides (a consensus ERE and a non-related probe) were added as competitors. The sequence of the probe containing the consensus ERE is 5'-CGGGCCGAGGTCACAGTGACCTCGGCCCGT-3' and the sequence of the non-related probe is 5'-CTAGTCCTAGGTCTAGAGAATTCA-3'.

### Cell culture and transfections

Human embryonic kidney 293 cell culture and transfections using Lipofectamine Plus reagent (Invitrogen) were done according to the manufacturer's recommendations and as previously described [94]. Briefly, 200 ng of the chimeras comprising the GAL4 DNA-binding domain fused with the LBD of either human ERα, lampER or AmphiER (or LBD for the control) were co-transfected together with 100 ng of reporter plasmid and 10 ng of a β-galactosidase expression vector, included as a control for transfection efficiency. For the mammalian double hybrid assays, the GAL4-amphiER-LBD chimera was transfected with 200 ng of the coactivator SRC1 fused to the strong activation domain VP16. Three to five hours post-transfection, serum and hormones (as indicated in the figures) were added to the cells which were incubated for an additional 48 hours before harvest and luciferase and β-galactosidase activities were determined. Results show the mean ± s.e.m. (n = 3) of representative experiments. Human HeLa cervical cancer cells and CV-1 green monkey kidney cells were routinely maintained in Dulbecco's modified eagle's medium (Invitrogen), supplemented with 10% fetal bovine seum, 1% v/v L-glutamine and 1% v/v penicillin/streptomycin. Cells were seeded in 12 or 24 well plates one day prior to transfection. Transient transfections were carried out using the Lipofectamine Plus reagent according to instructions of the manufacturer (Invitrogen) in culture media devoid of serum, phenol-red and antibiotics. Briefly, 1 ng of ERα, ERβ or AmphiER expression vectors were co-transfected together with 100 ng 3xERE-Luc (or 200 ng pS2-Luc where indicated) and 20 ng of a β-galactosidase expression vector, included as a control for transfection efficiency. In the co-expression experiments, AmphiER was co-transfected together with ERα or ERβ in ratios of 0.5:1, 1:1 and 1:5, respectively. Three hours post-transfection, serum and hormones (as indicated in the figures) were added to the cells which were thereafter incubated for an additional 48 hours before harvest and luciferase and β-galactosidase activities were determined. Figures represent results from at least three independent experiments performed in duplicates. Data is presented as mean +/- SD of fold induction of relative luciferase values corrected against β-galactosidase activity, where activity obtained from transfected reporter plasmid alone and treated with vehicle, was arbitrarily set to 1.

### Limited proteolytic digestion

These assays were done as previously described [13].

## Authors' contributions

MP, HE, MS, SB and VL contributed to the conception and design of the study. MS cloned amphiER. MP performed the EMSA, limited proteolysis experiments, part of the transactivaton assays and the bioinformatics study. KP and IP performed the rest of the transactivation assays. MP and VL wrote the manuscript.

## Supplementary Material

Additional file 1 **Phylogenetic analysis of NR3 sequences using several methods**. Phylogenetical trees of an alignment comprising 69 NR3 sequences as well as RXR sequences were inferred using the maximum likelihood method (ML) (A), Bayesian analysis (B), neighbour-joining method (C) and maximum parsimony method (MP) (D) based on an elision alignment of the DBD and LBD of 77 NR3 and RXRs (accession numbers are given in Additional file 8). Labels above each branch show percentages of bootstrap values after 1000 replicates (A), posterior probabilities (B), percentages of bootstrap values after 500 replicates (C) or 100 replicates (D). The fastest evolving sites (with an evolutionary rape above 2, as indicated in the Figure 3A) were removed from the alignment before computing phylogeny by maximum parsimony, to preserve the branching of mollusk ERs within the ER clade. In (A) nodes with bootstrap values below 50% are presented as polytomies, as in the Figure 2B. Click here for file

Additional file 2 **DNA binding characterization of chordate ERs**. Various chordate members of the NR3 family, namely human ERα, human ERβ, mouse ERRα, amphiER and lamprey ER, were synthesized *in vitro *and allowed to bind to a ^32^P-labeled consensus ERE probe in an EMSA. Lane 1, empty vector (pSG5) reticulocytes lysates. Lanes 2–5, human ERα. Lanes 6–9, human ERβ. Lanes 10–13, mouse ERRα. Lanes 14–17, amphiER. Lanes 18–21, lamprey ER. Lanes 3–5, 7–9, 11–13, 15–17, 19–21, unlabeled non-specific oligonucleotide (NS) or ERE were added at indicated molar excess as competitors to test the specificity of the binding. The arrows indicated the gel shift induced by amphiER binding the ERE probe. The asterisk indicates free ERE probe. Click here for file

Additional file 3 **The amphioxus ER acts as a dominant negative estrogen receptor in CV1 cells**. A pSG5 construct containing human ERα (A) or human ERβ (B) was tested in transfected CV1 cells for its ability to activate the co-transfected cognate ERE-luc reporter plasmid after E_2_, genistein or β-Androstane-diol stimulation (10^-6^M) in presence of increasing doses of the amphiER construct. Click here for file

Additional file 4 **The amphioxus ER is not activated by BPA**. (A) GAL4-LBD constructs from several chordate ERs were tested in transfected 293 cells for their ability to activate a (17 m)5x-G-luc reporter plasmid in the presence of increasing doses of BPA (10^-9^M to 10^-6^M). (B) Representation of the mammalian two-hybrid SRC1 recruitment assay. The GAL4-amphiER-LBD chimera was used with the coactivator SRC1 fused to the strong activation domain VP16 to transfect 293 cells in the presence of increasing doses of BPA (10^-9^M to 10^-6^M). Click here for file

Additional file 5 **amphiER is not activated by cholesterol derivatives**. (A) The GAL4-amphiER-LBD chimera was tested in transfected 293 cells for its ability to activate a (17 m)5x-G-luc reporter plasmid in the presence of various cholesterol derivatives at a high concentration (1 μM) (black). The empty vector (white) was used as a negative control and the GAL4-humanERα-LBD in the presence of E_2 _was used as a positive control (B) Representation of the mammalian two-hybrid SRC1 recruitment assay. The GAL4-amphiER-LBD chimera was used with the coactivator SRC1 fused to the strong activation domain VP16 to transfect 293 cells in the presence of various cholesterol derivatives at 1 μM. The empty vector (white) was used as a negative control. Click here for file

Additional file 6 **Limited proteolysis of amphiER with various cholesterol derivatives**. lane 1: undigested protein, lanes 2–4, 5–7: digested protein in the absence (lane 2 and 5) or presence (lanes 3–4 and 6–7) of ligand (10^-3^M and 10^-4^M). 2 different trypsine doses are shown, indicated by thick or thin bars above each panel. The ligands are cholic acid (A), Chenodeoxycholic acid (B), 22R-OH-cholesterol (C), cholesterol (D), 4-androstene-3,17-dione (E), DHEA (F), corticosterone (G), progesterone (H), pregnenolone (I), estrone (J), testosterone (K), 5α-androstane-dione (L), 20-hydroxyecdysone (M) and calcitriol (N). Click here for file

Additional file 7 **Phylogenetic tree of NR3 sequences as well as ancestral sequences**. Complete tree corresponding to the simplified one presented in the figure 7. The ancestral sequence of ER and NR3C was inferred using either a complete dataset (AncSRa) or a partial dataset (AncSRb) where 5 mollusk ER sequences as well as amphiER and amphiNR3C were omitted. The position of those sequences within the phylogenetic tree calculated with the complete dataset was compared. The position of a previously described ancestor (AncSR1) is indicated as well. Minimum of Chi2-based and SH-like supports are shown for each branch. Click here for file

Additional file 8 **Accession number of sequences used for phylogenetic analyses**. AR: androgen receptor; ER: estrogen receptor; ERR: estrogen related receptor; GR: glucocorticoid receptor; MR: mineralocorticoid receptor; PR: progesterone receptor; RXR: retinoid × receptor. Click here for file
