# Supplementary figures and images for: An amphioxus orthologue of the estrogen receptor that does not bind estradiol: Insights into estrogen receptor evolution
Source: BMC Evol Biol. 2008 Jul 25;8:219. doi: 10.1186/1471-2148-8-219 (PMC2529310; doi:10.1186/1471-2148-8-219)

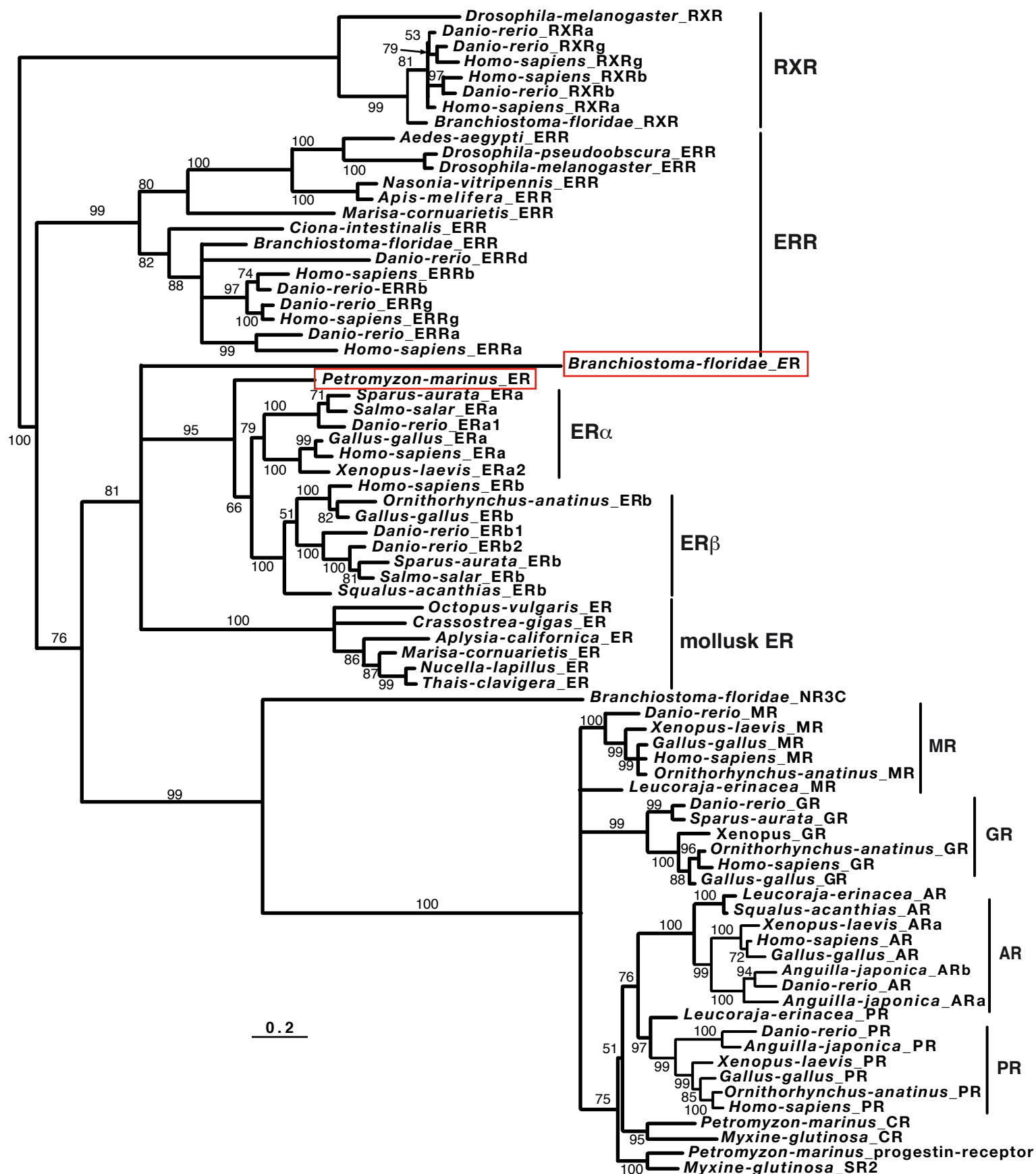

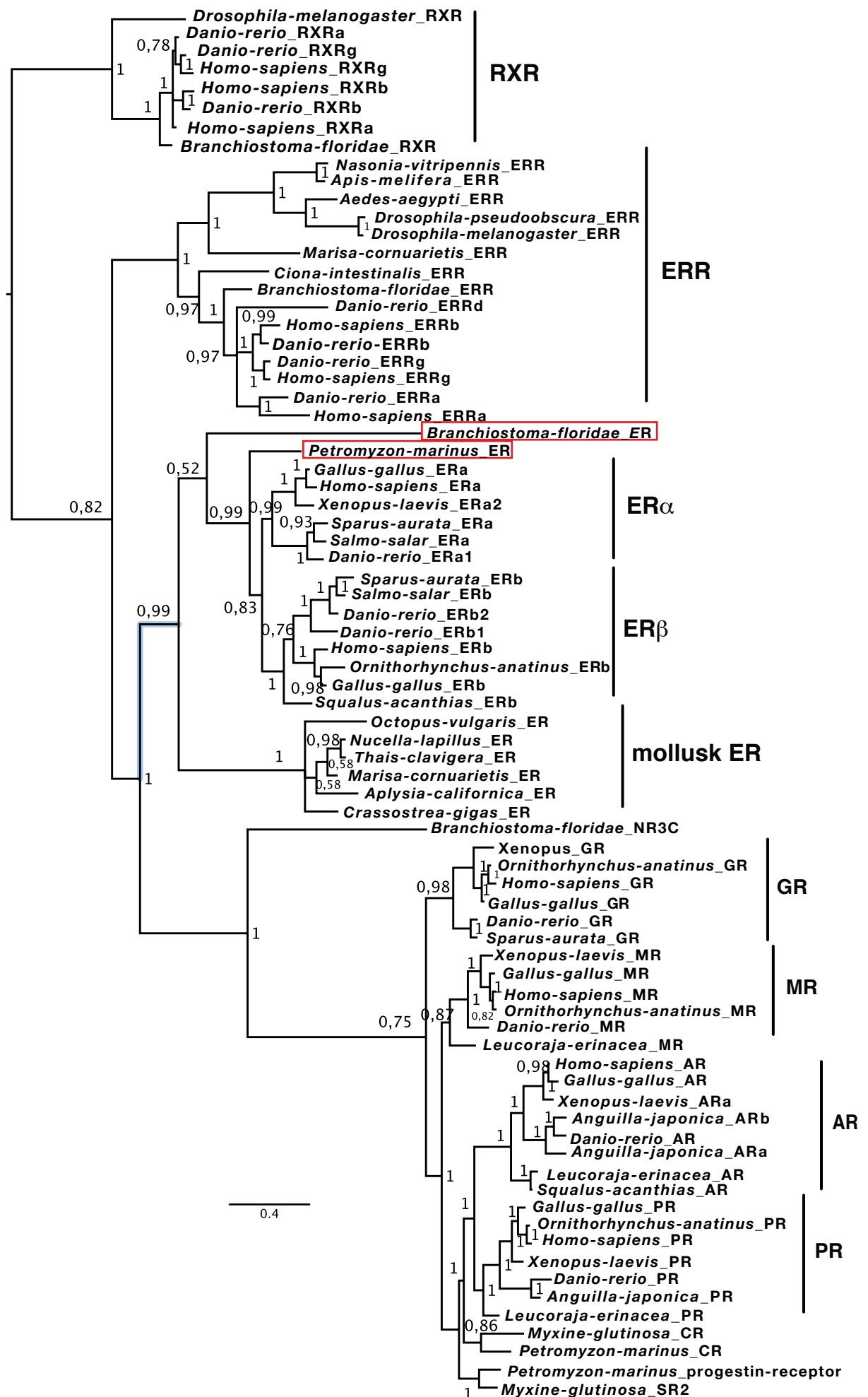

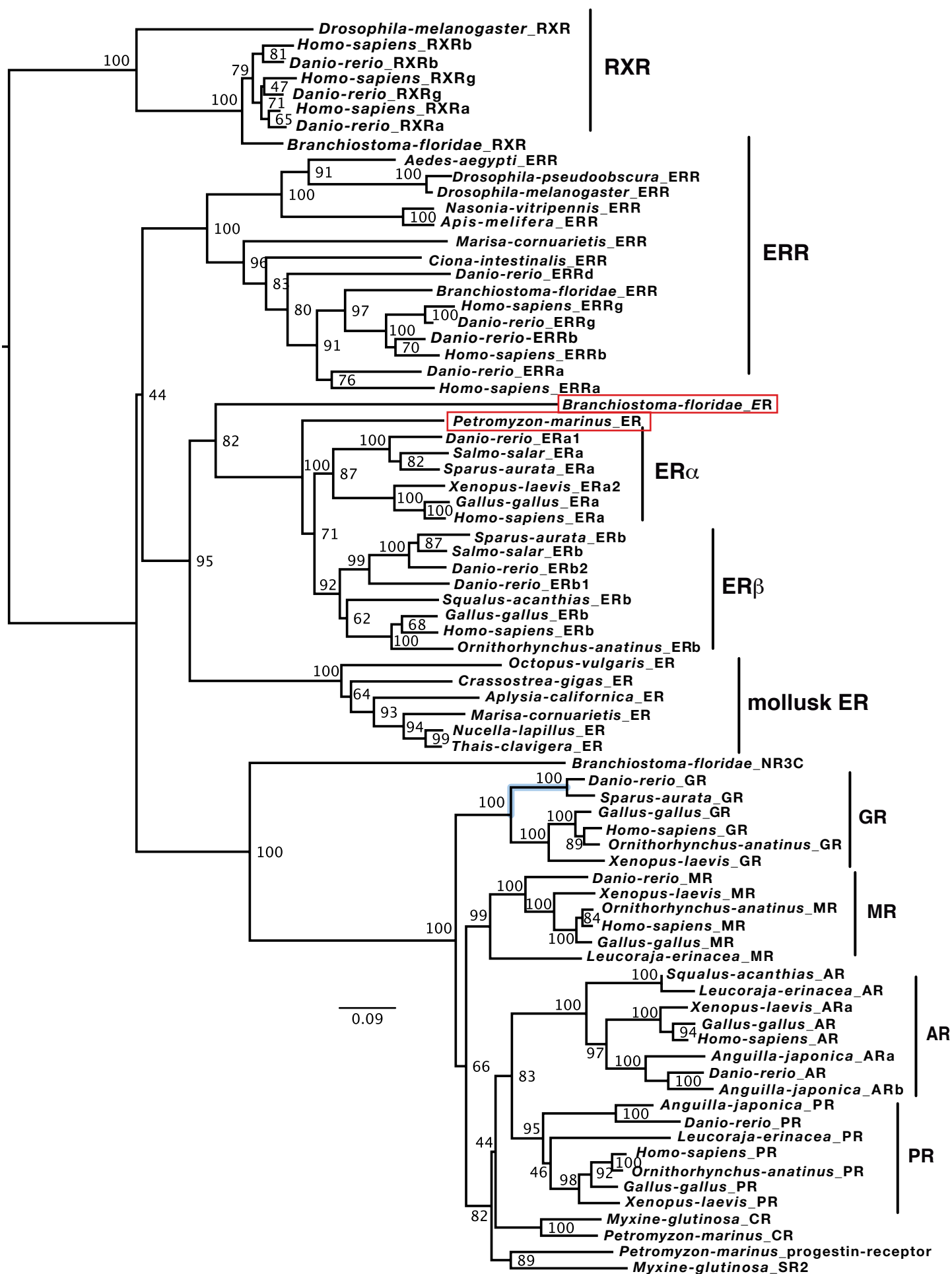

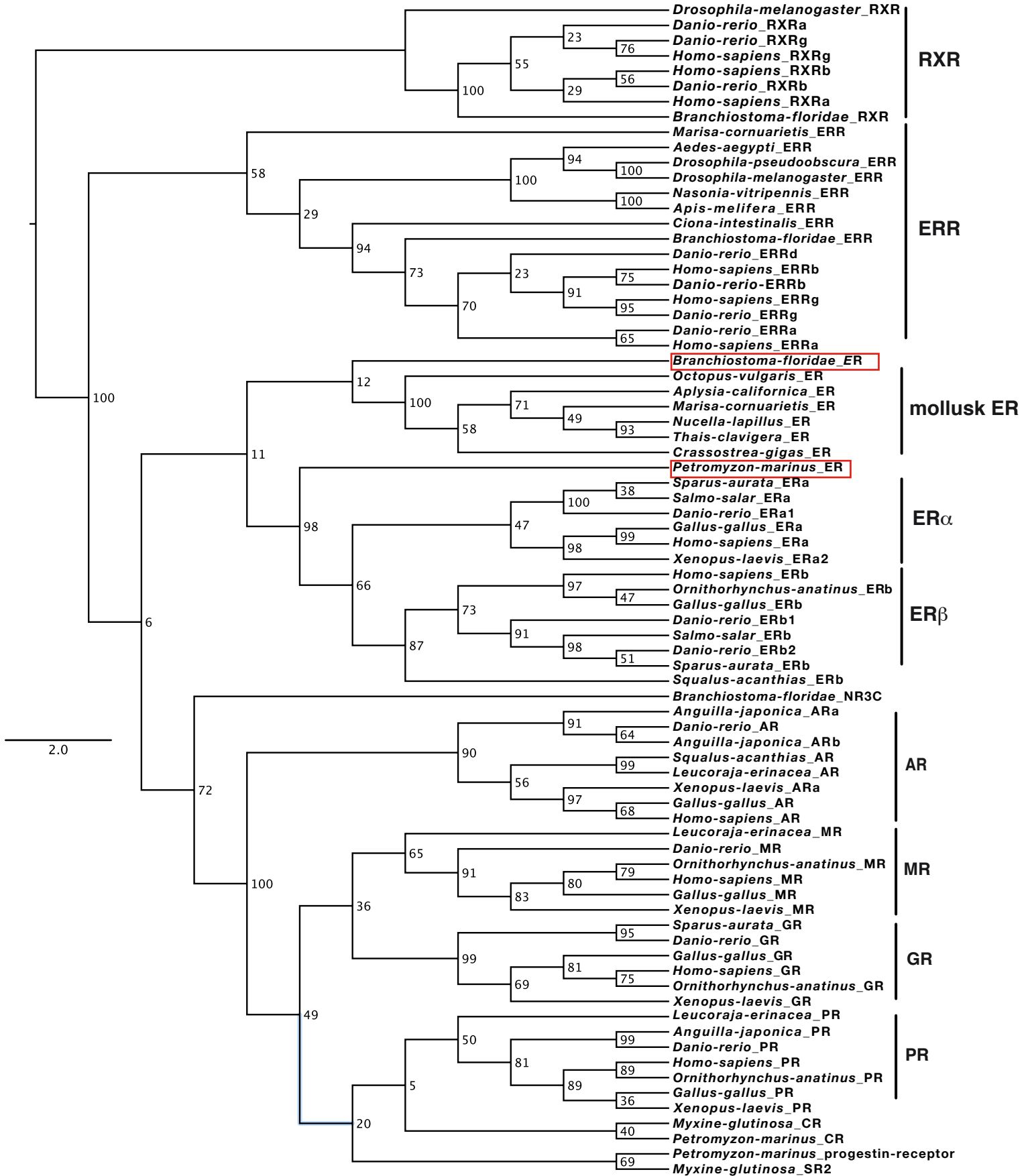

Supplement: Additional file 1 — Phylogenetic analysis of NR3 sequences using several methods. Phylogenetical trees of an alignment comprising 69 NR3 sequences as well as RXR sequences were inferred using the maximum likelihood method (ML) (A), Bayesian analysis (B), neighbour-joining method (C) and maximum parsimony method (MP) (D) based on an elision alignment of the DBD and LBD of 77 NR3 and RXRs (accession numbers are given in Additional file 8). Labels above each branch show percentages of bootstrap values after 1000 replicates (A), posterior probabilities (B), percentages of bootstrap values after 500 replicates (C) or 100 replicates (D). The fastest evolving sites (with an evolutionary rape above 2, as indicated in the Figure 3A) were removed from the alignment before computing phylogeny by maximum parsimony, to preserve the branching of mollusk ERs within the ER clade. In (A) nodes with bootstrap values below 50% are presented as polytomies, as in the Figure 2B. [file 1471-2148-8-219-S1.pdf]

human ER $\alpha$ human ER $\beta$ mouse ERR $\alpha$ 

amphioxus ER

lamprey ER

ERE  
NS

|   |   |     |    |     |   |     |    |     |   |     |    |     |   |     |    |     |   |     |    |     |
|---|---|-----|----|-----|---|-----|----|-----|---|-----|----|-----|---|-----|----|-----|---|-----|----|-----|
| - | - | -   | 10 | 100 | - | -   | 10 | 100 | - | -   | 10 | 100 | - | -   | 10 | 100 | - | -   | 10 | 100 |
| - | - | 100 | -  | -   | - | 100 | -  | -   | - | 100 | -  | -   | - | 100 | -  | -   | - | 100 | -  | -   |

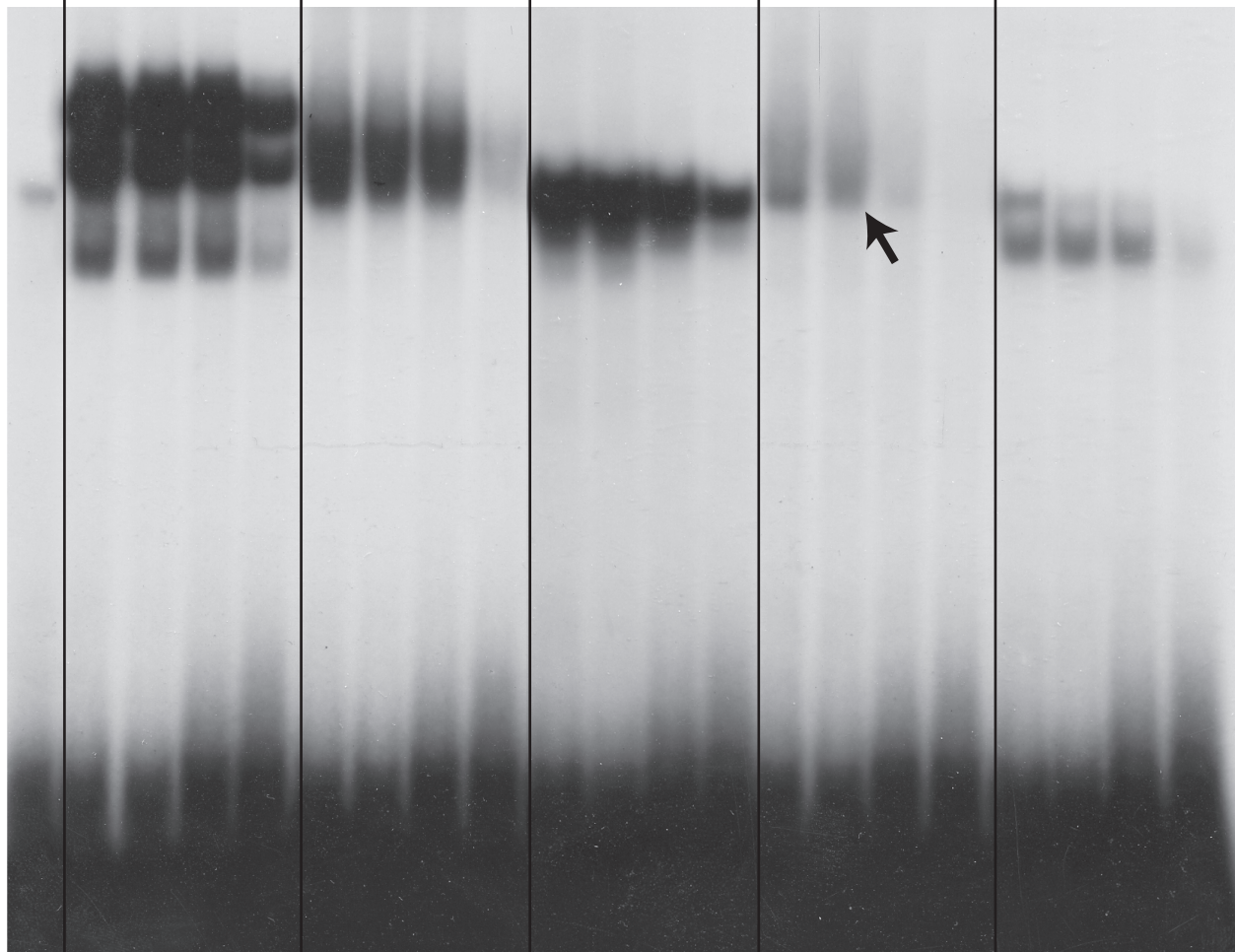

1 2 3 4 5 6 7 8 9 10 11 12 13 14 15 16 17 18 19 20 21

\*

Supplement: Additional file 2 — DNA binding characterization of chordate ERs. Various chordate members of the NR3 family, namely human ERα, human ERβ, mouse ERRα, amphiER and lamprey ER, were synthesized in vitro and allowed to bind to a 32P-labeled consensus ERE probe in an EMSA. Lane 1, empty vector (pSG5) reticulocytes lysates. Lanes 2–5, human ERα. Lanes 6–9, human ERβ. Lanes 10–13, mouse ERRα. Lanes 14–17, amphiER. Lanes 18–21, lamprey ER. Lanes 3–5, 7–9, 11–13, 15–17, 19–21, unlabeled non-specific oligonucleotide (NS) or ERE were added at indicated molar excess as competitors to test the specificity of the binding. The arrows indicated the gel shift induced by amphiER binding the ERE probe. The asterisk indicates free ERE probe. [file 1471-2148-8-219-S2.pdf]

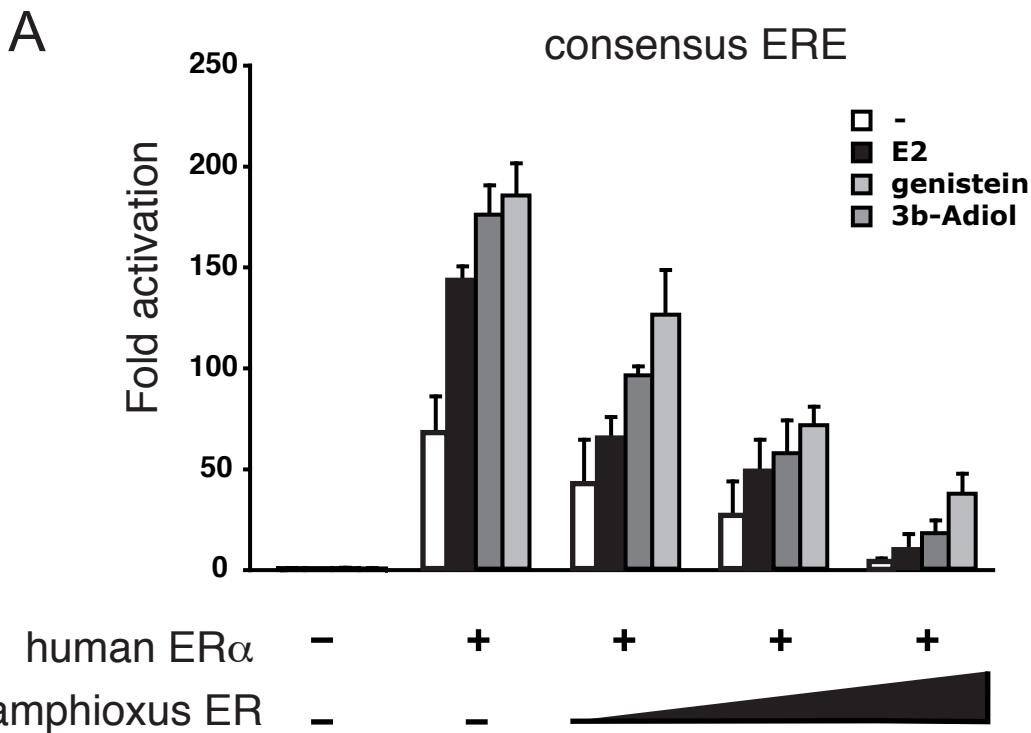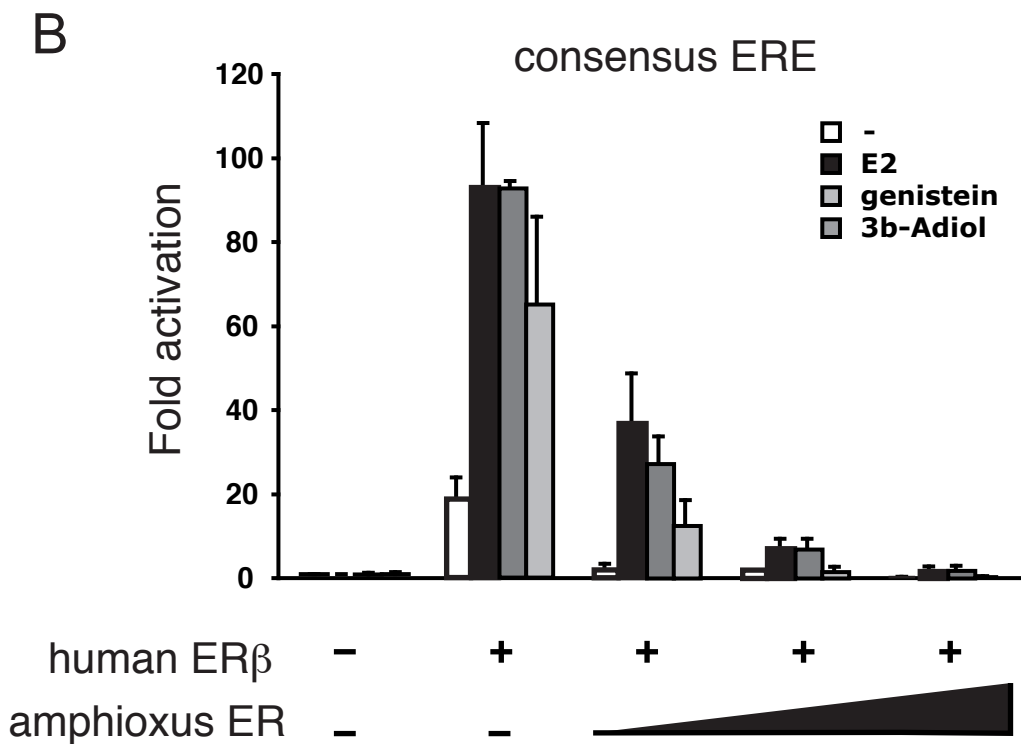

Supplement: Additional file 3 — The amphioxus ER acts as a dominant negative estrogen receptor in CV1 cells. A pSG5 construct containing human ERα (A) or human ERβ (B) was tested in transfected CV1 cells for its ability to activate the co-transfected cognate ERE-luc reporter plasmid after E2, genistein or β-Androstane-diol stimulation (10-6M) in presence of increasing doses of the amphiER construct. [file 1471-2148-8-219-S3.pdf]

A

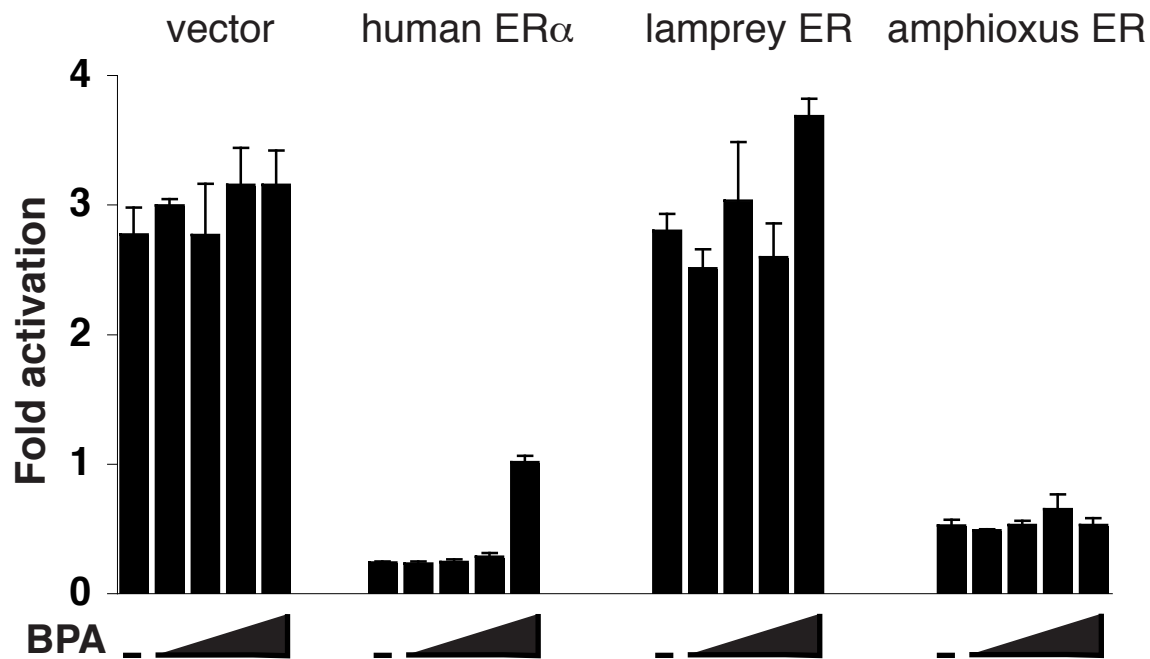

B

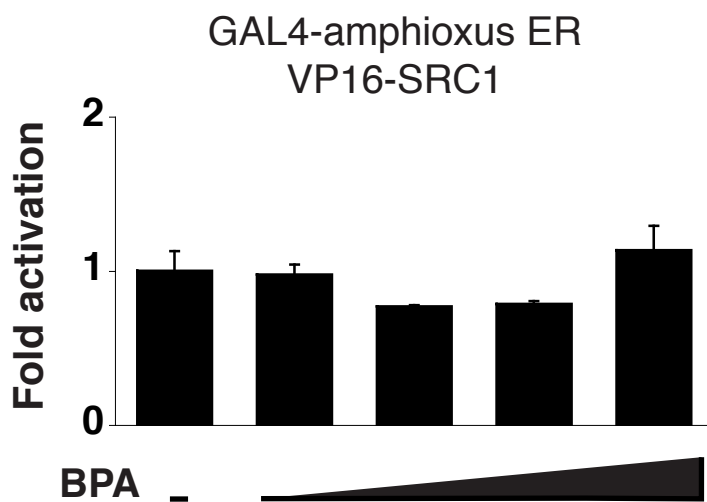

Supplement: Additional file 4 — The amphioxus ER is not activated by BPA. (A) GAL4-LBD constructs from several chordate ERs were tested in transfected 293 cells for their ability to activate a (17 m)5x-G-luc reporter plasmid in the presence of increasing doses of BPA (10-9M to 10-6M). (B) Representation of the mammalian two-hybrid SRC1 recruitment assay. The GAL4-amphiER-LBD chimera was used with the coactivator SRC1 fused to the strong activation domain VP16 to transfect 293 cells in the presence of increasing doses of BPA (10-9M to 10-6M). [file 1471-2148-8-219-S4.pdf]

A

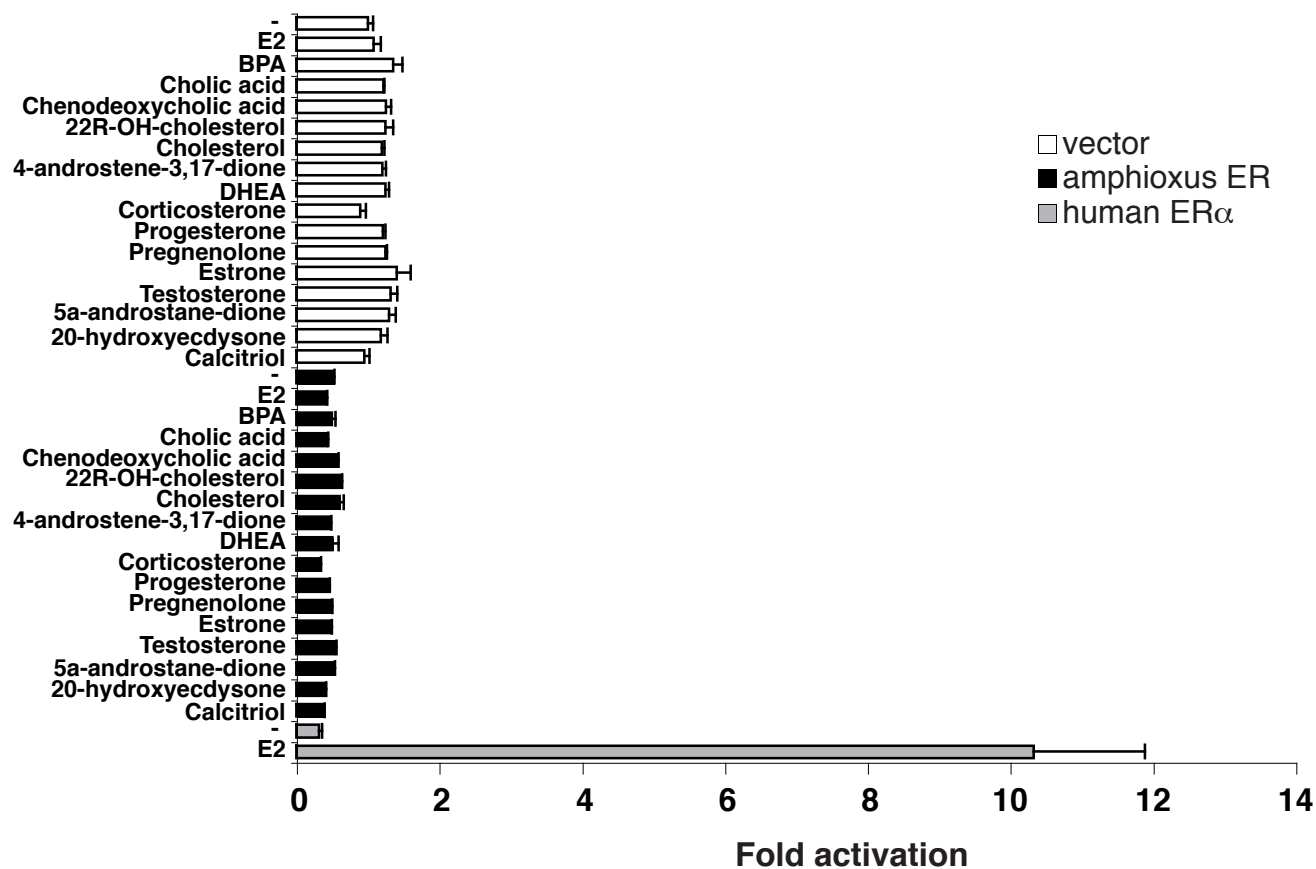

B

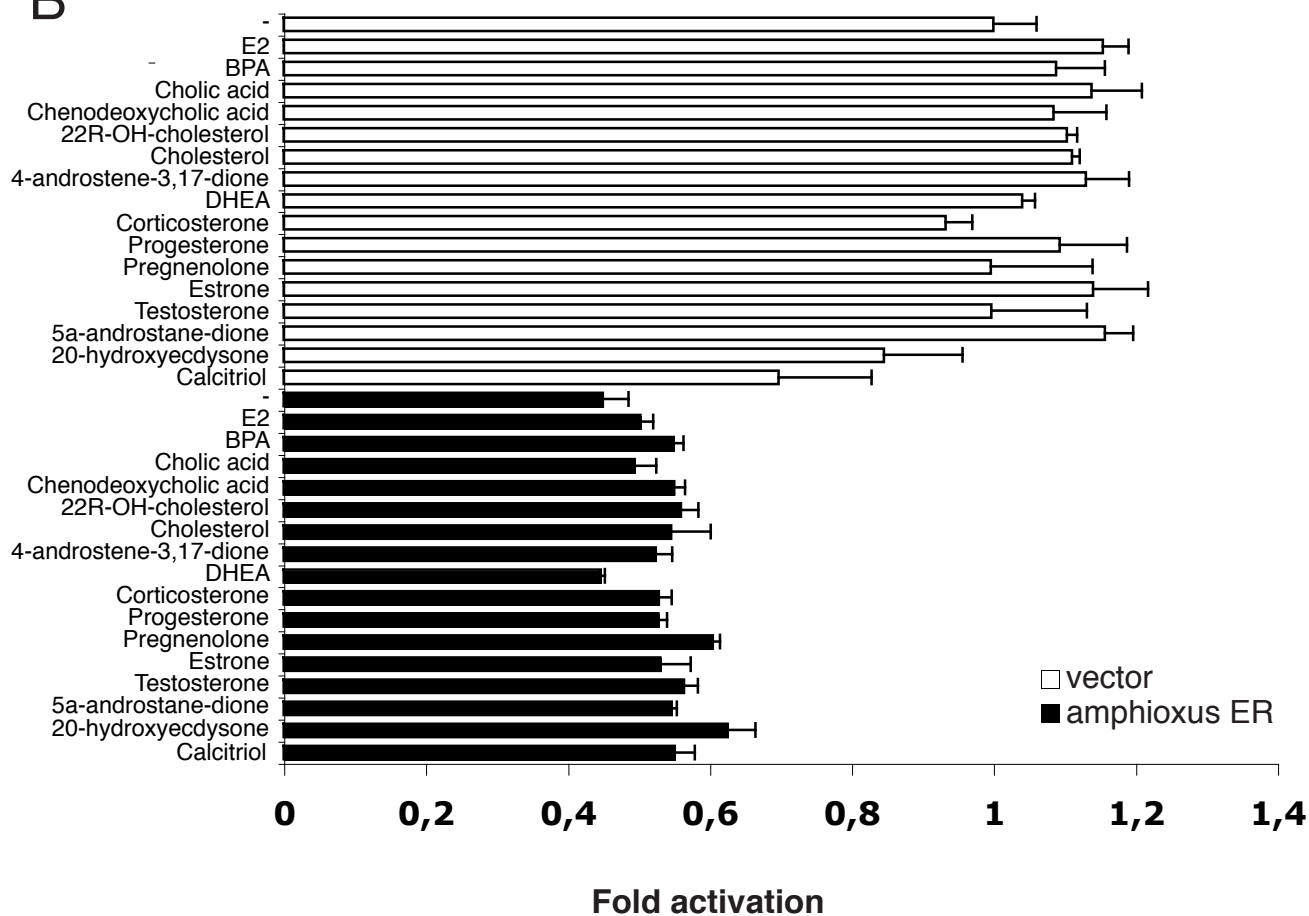

Supplement: Additional file 5 — amphiER is not activated by cholesterol derivatives. (A) The GAL4-amphiER-LBD chimera was tested in transfected 293 cells for its ability to activate a (17 m)5x-G-luc reporter plasmid in the presence of various cholesterol derivatives at a high concentration (1 μM) (black). The empty vector (white) was used as a negative control and the GAL4-humanERα-LBD in the presence of E2 was used as a positive control (B) Representation of the mammalian two-hybrid SRC1 recruitment assay. The GAL4-amphiER-LBD chimera was used with the coactivator SRC1 fused to the strong activation domain VP16 to transfect 293 cells in the presence of various cholesterol derivatives at 1 μM. The empty vector (white) was used as a negative control. [file 1471-2148-8-219-S5.pdf]

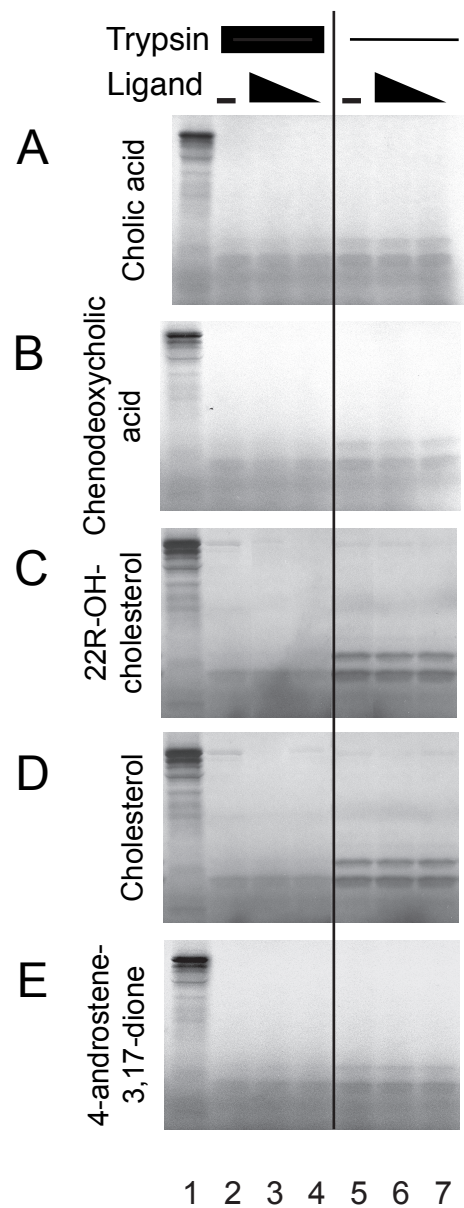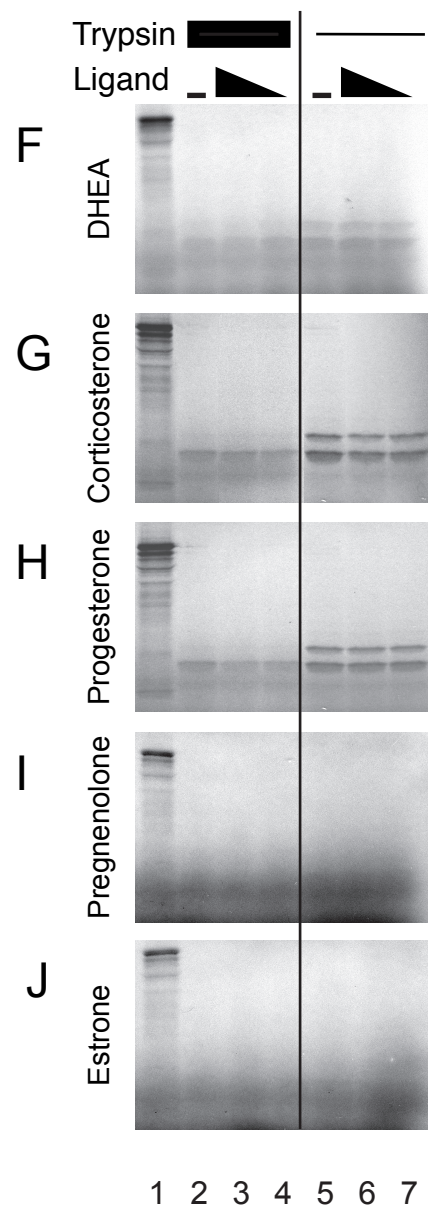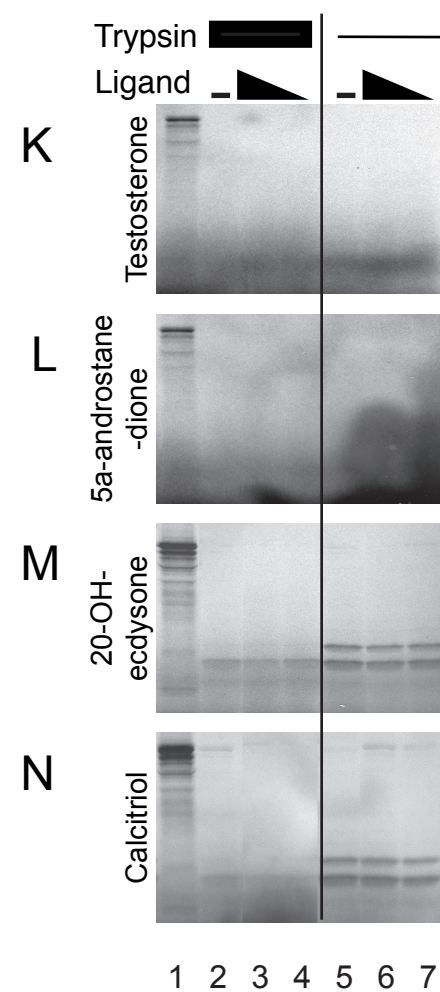

Supplement: Additional file 6 — Limited proteolysis of amphiER with various cholesterol derivatives. lane 1: undigested protein, lanes 2–4, 5–7: digested protein in the absence (lane 2 and 5) or presence (lanes 3–4 and 6–7) of ligand (10-3M and 10-4M). 2 different trypsine doses are shown, indicated by thick or thin bars above each panel. The ligands are cholic acid (A), Chenodeoxycholic acid (B), 22R-OH-cholesterol (C), cholesterol (D), 4-androstene-3,17-dione (E), DHEA (F), corticosterone (G), progesterone (H), pregnenolone (I), estrone (J), testosterone (K), 5α-androstane-dione (L), 20-hydroxyecdysone (M) and calcitriol (N). [file 1471-2148-8-219-S6.pdf]

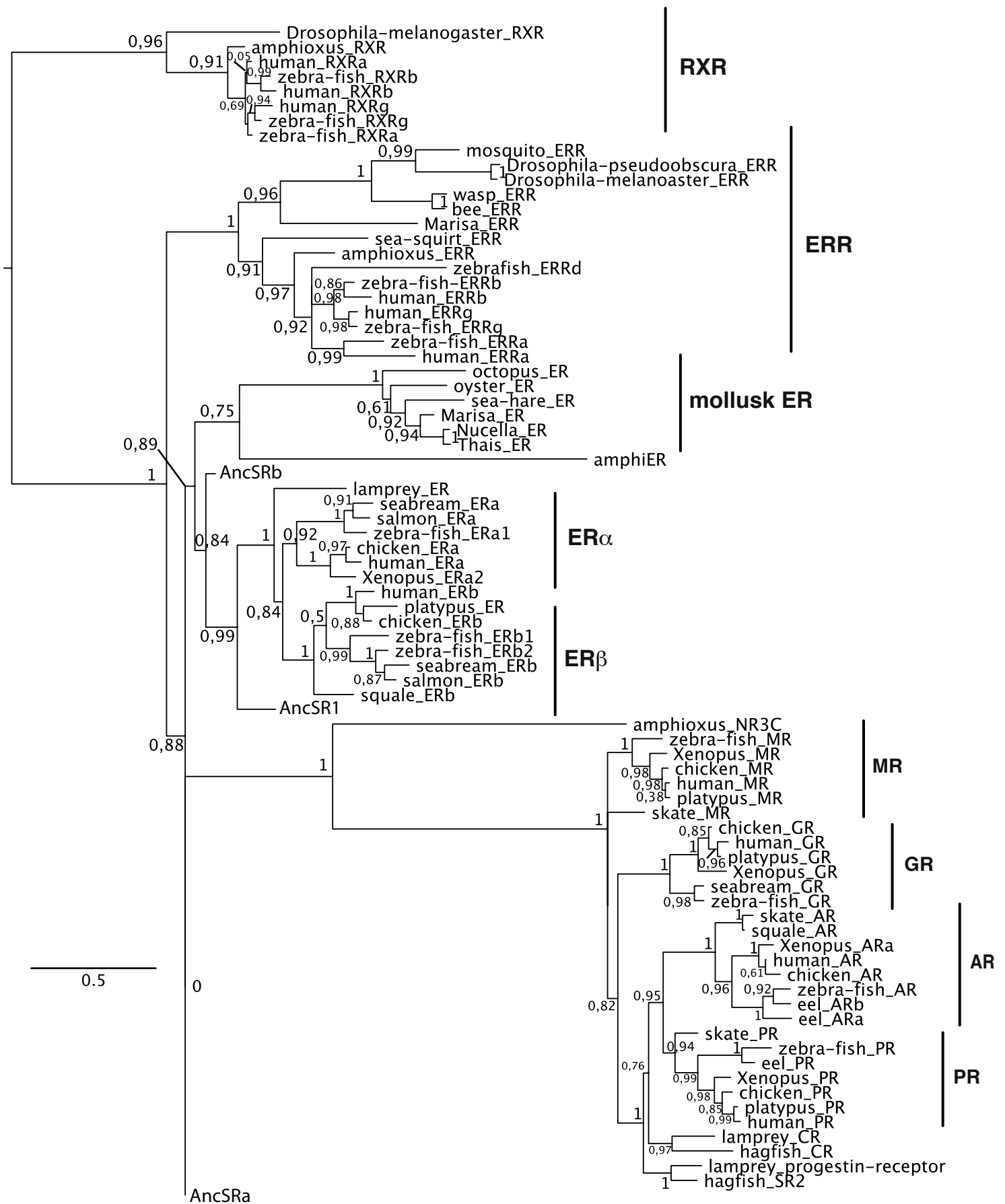

Supplement: Additional file 7 — Phylogenetic tree of NR3 sequences as well as ancestral sequences. Complete tree corresponding to the simplified one presented in the figure 7. The ancestral sequence of ER and NR3C was inferred using either a complete dataset (AncSRa) or a partial dataset (AncSRb) where 5 mollusk ER sequences as well as amphiER and amphiNR3C were omitted. The position of those sequences within the phylogenetic tree calculated with the complete dataset was compared. The position of a previously described ancestor (AncSR1) is indicated as well. Minimum of Chi2-based and SH-like supports are shown for each branch. [file 1471-2148-8-219-S7.pdf]
