# Supplementary material for: An amphioxus orthologue of the estrogen receptor that does not bind estradiol: Insights into estrogen receptor evolution
Source: BMC Evol Biol. 2008 Jul 25;8:219. doi: 10.1186/1471-2148-8-219 (PMC2529310; doi:10.1186/1471-2148-8-219)
Supplement: Additional file 8 — Accession number of sequences used for phylogenetic analyses. AR: androgen receptor; ER: estrogen receptor; ERR: estrogen related receptor; GR: glucocorticoid receptor; MR: mineralocorticoid receptor; PR: progesterone receptor; RXR: retinoid × receptor. [file 1471-2148-8-219-S8.pdf]

Accession number	Receptor name	*Species name*
AAA51729	AR	*Homo sapiens*
NP_001076592	AR	*Danio rerio*
ABD46746	AR	*Leucoraja erinacea*
AAP55843	AR	*Squalus acanthias*
NP_001035179	AR	*Gallus gallus*
NP_001084353	ARa	*Xenopus laevis*
BAA75464	ARa	*Anguilla japonica*
BAA83805	ARb	*Anguilla japonica*
ABD46742	CR	*Myxine glutinosa*
AAK20930	CR	*Petromyzon marinus*
ACF16007	ER	*Branchiostoma floridae*
BAF45381	ER	*Crassostrea gigas*
ABQ96884	ER	*Nucella lapillus*
ABI97119	ER	*Marisa cornuarietis*
ABG00286	ER	*Octopus vulgaris*
AAK20929	ER	*Petromyzon marinus*
AAQ95045	ER	*Aplysia californica*
BAC66480	ER	*Thais clavigera*
NP_990514	ERa	*Gallus gallus*
CAB51479	ERa	*Sparus aurata*
CAA27284	ERa	*Homo sapiens*
AAY25396	ERa	*Salmo salar*
NP_001083084	ERa2	*Xenopus laevis*
BAB16893	ERa1	*Danio rerio*
NP_990125	ERb	*Gallus gallus*
AAD31033	ERb	*Sparus aurata*
BAA24953	ERb	*Homo sapiens*
XP_001511889	ERb	*Ornithorhynchus anatinus*
AAK57823	ERb	*Squalus acanthias*
AAR92486	ERb	*Salmo salar*
CAC93848	ERb1	*Danio rerio*
CAC93849	ERb2	*Danio rerio*
XP_001663736	ERR	*Aedes aegypti*
AAU88062	ERR	*Branchiostoma floridae*
ABI97120	ERR	*Marisa cornuarietis*
NP_001071700	ERR	*Ciona intestinalis*
XP_392385	ERR	*Apis mellifera*
AAF50473	ERR	*Drosophila melanogaster*
XP_001604033	ERR	*Nasonia vitripennis*
CAA35778	ERRa	*Homo sapiens*
NP_998120	ERRa	*Danio rerio*
AAC99409	ERRb	*Homo sapiens*
AAS66635	ERRb	*Danio rerio*
XP_690815	ERRd	*Danio rerio*
AAC39899	ERRg	*Homo sapiens*
NP_998119	ERRg	*Danio rerio*
XP_001354210	ERR	*Drosophila pseudoobscura*

P49844	GR	*Xenopus laevis*
XP_001510749	GR	*Ornithorhynchus anatinus*
CAA26976	GR	*Homo sapiens*
ABS00394	GR	*Danio rerio*
ABF30967	GR	*Sparus aurata*
NP_001032915	GR	*Gallus gallus*
Q91573	MR	*Xenopus laevis*
AAA59571	MR	*Homo sapiens*
NP_001093873	MR	*Danio rerio*
XP_001513606	MR	*Ornithorhynchus anatinus*
ABD46745	MR	*Leucoraja erinacea*
XP_420437	MR	*Gallus gallus*
201600	NR3C	*Branchiostoma floridae (http://genome.jgi-psf.org/cgi-bin/dispGeneModel?db=Brafl1&tid=201600)*
AAY85275	PR	*Danio rerio*
AAA60081	PR	*Homo sapiens*
NP_990593	PR	*Gallus gallus*
NP_001079100	PR	*Xenopus laevis*
XP_001510439	PR	*Ornithorhynchus anatinus*
ABD46747	PR	*Leucoraja erinacea*
BAA89539	PR	*Anguilla japonica*
AAK20931	progestin-receptor	*Petromyzon marinus*
CAA37496	RXR	*Drosophila melanogaster*
AAM46151	RXR	*Branchiostoma floridae*
CAA36982	RXRa	*Homo sapiens*
AAC59719	RXRa1	*Danio rerio*
AAA60293	RXRb	*Homo sapiens*
AAC59722	RXRb1	*Danio rerio*
AAA80681	RXRg	*Homo sapiens*
AAC59720	RXRg1	*Danio rerio*
ABD46743	SR2	*Myxine glutinosa*
AAQ98788	AncSR1 (DBD)	*Ancestral sequence of steroid receptors*
AAQ98789	AncSR1 (LBD)	*Ancestral sequence of steroid receptors*
